# Modulation of Fructose Transfer Process for Promoting Apparent Isomerization Activity of Amylosucrase from *Bifidobacterium thermophilum*

**DOI:** 10.1021/acs.jafc.6c02580

**Published:** 2026-04-06

**Authors:** Yoon-Ji Jeong, Dong-Ho Seo, Sang-Ho Yoo

**Affiliations:** Department of Food Science & Biotechnology and Carbohydrate Bioproduct Research Center, 35006 Sejong University, 209 Neungdong-ro, Gwangjin-gu, Seoul 05006, Republic of Korea

**Keywords:** amylosucrase, site-saturation mutation, turanose, trehalulose

## Abstract

Amylosucrase catalyzes the synthesis of valuable carbohydrate products, including α-1,4 glucans and sucrose isomers, but controlling product distribution remains challenging. We engineered the +2 subsite of *Bifidobacterium thermophilum* amylosucrase through saturation mutagenesis at Gly374 to enhance product selectivity. Among 19 variants, G374H and G374T demonstrated remarkable improvements in turanose and trehalulose production, respectively. G374H exhibited a 9.1-fold increase in catalytic efficiency for turanose formation, achieving 88.64% selectivity. G374T showed a 7.8-fold enhancement in trehalulose production efficiency. Molecular dynamics simulations revealed that these substitutions induced coordinated structural changes, specifically by modulating the flexibility of active-site loops (loops 3 and 7) and optimizing the local hydrogen-bond network, which collectively determine product specificity. G374H showed enhanced conformational flexibility for optimal substrate positioning, while G374T displayed efficient product release mechanisms. This work demonstrates that strategic modification of substrate-binding residues enables selective control of product distribution for functional food ingredient production.

## Graphical abstract

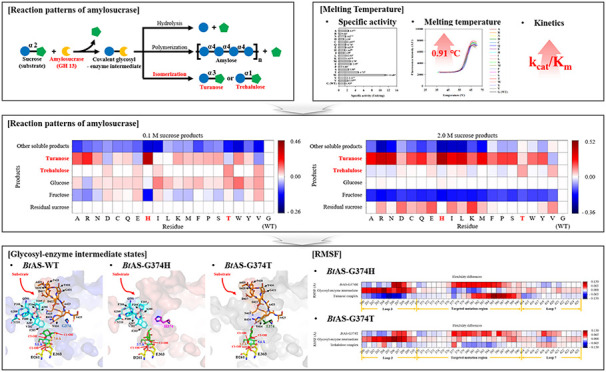

## 1 Introduction

Sucrose, a disaccharide composed of glucose and fructose linked by an α-1,2-glycosidic bond, represents one of the widely used sweeteners in the food industry.^1^ Despite its widespread use, concerns regarding its high glycemic index and cariogenic potential have prompted interest in alternative sweeteners. Sucrose isomers such as isomaltulose (palatinose), turanose, and trehalulose exhibit significant structural differences that lead to substantial variations in their biochemical and physicochemical properties, as well as their biological activities.^2,3^ These sucrose isomers confer advantages such as lower glycemic indices, reduced cariogenicity, and beneficial modulation of gut microbiota.^2,4^ Among these isomers, turanose (α-d-glucopyranosyl-(1→3)-d-fructose) and trehalulose (α-d-glucopyranosyl-(1→1)-d-fructose) have gained particular attention due to their unique structural properties and potential health benefits. Turanose demonstrates multiple promising applications, notably anti-inflammatory effects in colitis models, improved rheological properties in food systems, and effective cryoprotection for probiotic bacteria during freeze-drying processes.^5^ Similarly, trehalulose shows promise as an alternative sweetener and functional ingredient across various industries such as food, pharmaceuticals, and cosmetics, owing to its low-calorie content, low glycemic index, and antioxidant properties.^6^

Several production methods have been explored for the synthesis of these valuable sucrose isomers. Chemical synthesis methods for turanose production suffer from limited specificity and generate unwanted byproducts that necessitate extensive purification procedures.^7^ Enzymatic approaches using cyclodextrin glucanotransferase yield approximately 43% conversion, offering better specificity but still facing challenges in optimizing reaction conditions and overcoming substrate constraints.^2,8^ Another approach utilizes fermentation with microorganisms such as *Serratia plymuthica*, which produces turanose at approximately 35% yield, but encounters difficulties in downstream processing, purification, and scale-up to industrial levels.^9^ Among the enzymatic methods, amylosucrase (ASase, E.C. 2.4.1.4)-based production has emerged as a particularly promising approach for turanose synthesis, achieving significantly higher conversion yields under mild reaction conditions.^10–12^ ASase is a transglucosidase, a multifunctional enzyme that uses sucrose as a sole substrate to biosynthesize sucrose isomers such as turanose and trehalulose as well as α-1,4-glucan.^13^ Specifically, ASase produces sucrose isomers through an isomerization reaction that utilizes fructose as an acceptor molecule generated from initial hydrolysis (Figure S1). Initially, sucrose isomers were not observed during the initial ASase reaction. However, they began to form once fructose concentration accumulated to sufficient levels following sucrose hydrolysis.^4^ Previous studies have shown that when exogenous fructose was added to the ASase reaction with sucrose, turanose production increased while glucan synthesis decreased.^11,14,15^ This shift occurs because exogenous fructose competes as an acceptor molecule for the ASase-glucose complex, which forms when glucose from sucrose binds covalently to the enzyme, redirecting synthesis toward turanose rather than α-1,4-glucan.

The engineering of positive subsites (+1 to + n) has been established as a versatile strategy to modulate transglycosylation activity and acceptor specificity, not only in ASases but also across various Glycoside Hydrolase (GH) families.^16–19^ ASase contains multiple substrate binding subsites (-n to + n) within its active site region that play crucial roles in donor and acceptor recognition, binding specificity, and catalytic efficiency during transglycosylation reactions.^16,17,20^ Among these, the +2 subsite plays a particularly important role in substrate access to the active site, and structural variations in this region have been identified as critical determinants of the distinct transglycosylation activities observed across various ASases.^12,14,21^ Amino acid mutations at Gly396 and Asp394 within the +2 subsite of ASase from *Neisseria polysaccharea* (*Np*AS) restricted α-1,4-glucan chain elongation capability. These mutations generated variants that terminated transglycosylation reactions at disaccharide or trisaccharide levels due to diminished substrate binding and translocation capacities.^22^ Specifically, previous study demonstrated that the G396S mutation in *Np*AS substantially reduced polymerization activity, redirecting the enzyme’s catalytic function toward enhanced turanose production.^12^ Similarly, the analogous G374S mutation in ASase from *Bifidobacterium thermophilum* (*Bt*AS) showed a dramatic increase in turanose synthesis, confirming the crucial role of this conserved glycine residue in the +2 subsite for controlling product specificity across different ASases.^23^ Additionally, these studies established that supplementation with fructose as an exogenous acceptor substantially enhanced turanose yields, demonstrating the potential for engineered +2 subsite variants to optimize sucrose isomer production.^11,14,15^ In this study, site-saturation mutagenesis was performed at the G374 residue in the +2 subsite of *Bt*AS to systematically investigate how amino acid substitutions at this position affect sucrose isomer production specificity. We hypothesized that different amino acid properties at the *Bt*AS G374 position would modulate fructose binding orientation and consequently determine the type of glycosidic linkage formed. While previous studies focused on single mutations that enhanced turanose production, our comprehensive approach aimed to establish complete structure–function relationships and identify variants with tunable product selectivity. Through kinetic analysis and molecular dynamics simulations, this work aims to establish mechanistic principles for rational engineering of product-selective ASase variants.

## 2 Materials and Methods

### 2.1 ASase Gene, Bacterial Strain, and Growth Conditions

The ASase gene from *B. thermophilum* JCM 1207 (Gene locus tag: BTHE_RS02440, Protein ID: WP_044279707.1) was obtained as described in a previous study.^15^ *Escherichia coli* DH5α [*F-φ80d lacZΔM15 Δ­(lacZYA-argF) U169 end A1 recA1 hsdR17 (rk* ^–^, *mk* ^*+*^ *) supE44λ- thi-1 gyrA96relA1 phoA*], a cloning strain with high transformation efficiency and reduced recombination activity, was used for site-directed mutagenesis, plasmid propagation, and genetic manipulation. After verifying the mutated amino acid sequence, *E. coli* BL21 [*F* ^–^ *ompT hsdS­(rB* ^–^ *mB* ^–^ *) gal dcm­(DE3)*], an expression host containing the T7 RNA polymerase gene under the control of the lacUV5 promoter, was used for the inducible expression of wild-type and mutant *Bt*AS proteins using pET28a-based vectors. Both WT and variant forms of *Bt*AS were expressed and purified as previously described.^15^

### 2.2 Site-Saturation Mutagenesis of *Bt*AS

Based on prior structural and functional studies,^23^ Gly374 at the +2 subsite of *Bt*AS, known to influence sucrose isomer specificity, was selected for saturation mutagenesis to systematically explore its role in product selectivity.

Site-saturation mutagenesis at position Gly374 was performed using the QuickChange II Site-Directed Mutagenesis Kit (Agilent Technologies, Santa Clara, CA, USA) with pET28a-*Bt*AS as template. Forward and reverse primers were designed to replace the glycine codon (GGC) at position 374 with codons corresponding to all 19 amino acids (Table S1). PCR amplification was conducted using standard conditions: 25 cycles of denaturation (95 °C, 30 s), annealing (55 °C, 1 min), and extension (70 °C, 5 min). Wild-type (WT) DNA was removed by *Dpn*I digestion, and mutant plasmids were transformed into *E. coli* DH5α competent cells. Successful mutations were confirmed by DNA sequencing using T7 primers. All strains, plasmids, and primers used in this study are summarized in Supplementary Table S2.

### 2.3 Enzyme Expression and Purification

Mutant plasmids were transformed into *E. coli* BL21­(DE3) competent cells and expressed following standard protocols. Briefly, cells were cultured in LB medium containing 50 μg/mL kanamycin at 37 °C until OD_600_ reached 0.6–0.8. Protein expression was induced with 0.2 mM Isopropyl β-d-1-thiogalactopyranoside (IPTG) and cells were incubated at 18 °C for 20 h. Cells were harvested by centrifugation, resuspended in 50 mM Tris-HCl buffer (pH 7.0), and lysed by sonication. The (His)_6‑_tagged *Bt*AS variants were purified using Ni-NTA affinity chromatography (Qiagen, Hilden, Germany) and concentrated in 50 mM Tris-HCl buffer (pH 7.0). Protein purity was confirmed by SDS-PAGE analysis using standard protocols, and protein concentrations were determined by Bradford assay.

### 2.4 Biochemical Characterization of *Bt*AS Variants

#### 2.4.1 Enzyme Activity Assay

Enzyme activity was measured by quantifying reducing sugars released from sucrose hydrolysis using the DNS method.^21^ Reactions were performed in 50 mM sodium acetate buffer (pH 6.0) containing 0.1 M sucrose at 50 °C for 30 min. One unit of enzyme activity was defined as the amount of enzyme releasing 1 μmol of fructose per minute. Protein concentrations were determined by Bradford assay using bovine serum albumin as standard.

#### 2.4.2 Melting Temperature of Mutants

Apparent melting temperatures (*T*_*m,app*_) of *Bt*AS variants were determined by differential scanning fluorimetry using SYPRO Orange dye.^24^ Purified enzymes (0.3 mg/mL) were analyzed from 25 to 99 °C in 1 °C increments using a real-time PCR system.

#### 2.4.3 Product Analysis

All 19 *Bt*AS variants were screened for product formation compared to the WT enzyme. Reactions were performed in 50 mM sodium acetate buffer (pH 6.0) at 50 °C for 24 and 72 h using 0.3 mg/mL enzyme with either 0.1 M (low) or 2 M (high) sucrose concentrations. Products were analyzed by high-performance liquid chromatography coupled with evaporative light scattering detection (HPLC-ELSD) and high-performance anion-exchange chromatography with pulsed amperometric detection (HPAEC-PAD). The main products identified were glucose, fructose, sucrose, turanose, trehalulose, and other soluble oligosaccharides.

### 2.5 Detailed Characterization of *Bt*AS-G374H and *Bt*AS-G374T

#### 2.5.1 Temperature and pH Optimization

The optimal temperature and pH for *Bt*AS-G374H and *Bt*AS-G374T were determined using the DNS assay. Temperature effects were evaluated from 25 to 65 °C, and pH effects were assessed from pH 4–10 using appropriate buffer systems (sodium acetate, sodium phosphate, Tris-HCl, and glycine-NaOH buffers, 50 mM each).

#### 2.5.2 Kinetic Analysis

Kinetic parameters (*K*_*m*_ and *k*_*cat*_) for turanose and trehalulose production were determined by varying fructose concentrations (18–100 mM) while maintaining sucrose at 100 mM. Reactions were performed using 0.3 mg/mL enzyme for 30 min at optimal conditions. Products were quantified by HPAEC-PAD using a CarboPac PA1 column. All experiments were performed in triplicate. Catalytic efficiency (*k*_*cat*_/*K*_*m*_, mM^–1^ ·min^–1^) was calculated to assess enzymatic performance combining turnover rate and substrate affinity, while production efficiency was defined as the percentage of the target isomer relative to the total amount of isomerization products.

#### 2.5.3 Time-Course Monitoring of Sucrose Isomer Production

Product formation by *Bt*AS WT, G374H, and G374T was monitored over 72 h (0, 2, 4, 8, 12, 24, 36, 48, 60, 72 h) using 0.3 mg/mL enzyme and 2.0 M sucrose as sole substrate at optimal conditions.

### 2.6 HPLC-ELSD and HPAEC-PAD Analysis Conditions

Reaction products were analyzed by HPLC-ELSD and HPAEC-PAD after dilution and filtration through 0.2 μm filters. For HPLC-ELSD analysis, samples were separated using a VG-50–4E column (4.6 × 250 mm, 5 μm, Shodex, Tokyo, Japan) on an Agilent 1260 Infinity system (Agilent Technologies, Santa Clara, CA, USA) with a mobile phase consisting of methanol:water (1:2) and acetonitrile in gradient mode. The gradient profile was as follows: acetonitrile was held at 87% from 0 to 25 min, briefly decreased to 80%, then returned to 87% at 25.1 min and maintained until 30 min. The flow rate was maintained at 1.0 mL/min with an injection volume of 5 μL and column temperature of 60 °C. Detection was performed using an Alltech 2000 ELSD (Alltech Associates, Deerfield, IL, USA). HPAEC-PAD analysis was performed using a Dionex ICS-5000 system (Thermo Fisher Scientific, Waltham, MA, USA) with a CarboPac PA1 column (4 × 250 mm, Dionex, Sunnyvale, CA, USA). An isocratic elution with 100 mM NaOH was performed for 20 min, followed by a 5 min column washing step using 100 mM NaOH containing 600 mM sodium acetate. The flow rate was 1.0 mL/min and the injection volume was 20 μL. Detection was achieved using an ED40 electrochemical detector with pulsed amperometric detection (PAD; Dionex, Sunnyvale, CA, USA).

### 2.7 Structural Modeling and Molecular Dynamics Simulations

#### 2.7.1 Structure Prediction

Three-dimensional structures of *Bt*AS WT and its G374H and G374T variants were predicted using AlphaFold3 ^25^ under default settings. Point mutations were introduced into the amino acid sequence prior to structure prediction, and AlphaFold3 generated full-atom structures *de novo* based on the modified sequences. AlphaFold3 was also used to model ligand-bound states using its integrated ligand-aware prediction capability. SMILES representations of sucrose, fructose, turanose, and trehalulose were supplied as ligand inputs, enabling direct prediction of enzyme–ligand complexes without requiring separate molecular docking protocols. Three distinct molecular states were modeled for each enzyme variant: (1) enzyme–sucrose complex, (2) glycosyl-enzyme intermediate with docked fructose, and (3) enzyme–product complex with turanose or trehalulose. Specifically, to model the glycosyl-enzyme intermediate state, the covalent linkage between the glucose moiety and the catalytic nucleophile (Asp261) was explicitly specified based on the proposed catalytic mechanism (Figure S1). The fructose acceptor was simultaneously positioned relative to the anomeric carbon of the covalently bound glucose natively by AF3’s ligand-aware prediction. Structural confidence was assessed using per-residue pLDDT scores (>96), and interatomic distances within the active site were measured using PyMOL 3.1 (Schrödinger LLC, New York, NY, USA) to evaluate substrate positioning and catalytic potential.

#### 2.7.2 Molecular Dynamics Simulations

Molecular dynamics (MD) simulations were performed using GROMACS 2024.1 with the CHARMM36 all-atom force field.^26^ Enzyme-ligand complexes predicted by Alphafold3 (Section 2.7.1) were used directly as initial conformations. Each system was placed in a cubic simulation box and solvated with TIP3P water molecules, ensuring a minimum distance of 1.0 nm between the protein and box edges. Systems were neutralized and brought to 0.15 M ionic strength by adding Na^+^ and Cl^–^ ions. All ionizable residues were assigned standard protonation states using GROMACS pdb 2gmx default settings: aspartate and glutamate residues were deprotonated (negatively charged), lysine and arginine residues were protonated (positively charged), and histidine residues were assigned HSE (Nε-protonated) states. The catalytic residues Asp261 and Glu303 were maintained in their ionized states.

Ligand topologies and parameters for sucrose, fructose, turanose, and trehalulose were generated using the CHARMM General Force Field (CGenFF) server (https://cgenff.com) to ensure full compatibility with the CHARMM36 protein force field.

Following energy minimization using the steepest descent algorithm, systems underwent equilibration in two phases: 6 ns in the NVT ensemble at 300 K, followed by 6 ns in the NPT ensemble at 300 K and 1 bar. Production MD simulations were then conducted for 100 ns with a 2 fs time step. Temperature was maintained at 300 K using the V-rescale thermostat (τ = 0.1 ps), and pressure was maintained at 1 bar using the Parrinello–Rahman barostat (τ = 2.0 ps). Long-range electrostatic interactions were calculated using the Particle Mesh Ewald (PME) method with a cutoff of 1.2 nm, and all hydrogen bonds were constrained using the Linear Constraint Solver (LINCS) algorithm. Root mean square deviation (RMSD) and root-mean-square fluctuation (RMSF) were calculated to assess structural stability and conformational dynamics of active site loops.

#### 2.7.3 Statistical Analysis

All experiments were independently conducted in triplicate, and results are presented as the mean ± standard deviation (SD). Statistical analyses were carried out using SPSS software (version 12.0; IBM Corp., Armonk, NY, USA). To evaluate differences among multiple groups, one-way analysis of variance (ANOVA) was performed, followed by Tukey’s post hoc test for multiple comparisons. For two-group comparisons, the Student’s *t* test was applied. A *p*-value of less than 0.05 was considered statistically significant.

## 3 Results and Discussion

### 3.1 Construction and Expression of *Bt*AS Gly374 Variants

A complete set of 19 amino acid substitution variants was generated at the Gly374 position using site-saturation mutagenesis. Expression and purification of all variants were confirmed by SDS-PAGE analysis (Figure S2). All variants showed consistent expression levels and high purity with bands at approximately 70 kDa, consistent with the expected molecular weight of *Bt*AS.^15^ Specific activities of all variants were determined and compared with the WT enzyme (Table 1). The WT exhibited a specific activity of 1.52 U/mg, while the variants showed considerable variation in their catalytic activities. Notably, the G374W variant demonstrated remarkably enhanced specific activity (11.48 U/mg), representing a 7.6-fold increase compared to the WT. This enhanced specific activity suggests greater catalytic efficiency in both hydrolysis and isomerization reactions, potentially increasing the production of reducing sugars (glucose and fructose) as well as reducing sucrose isomers such as turanose and trehalulose. To assess protein stability, the apparent melting temperature (*T*_*m,app*_) of all variants was measured by DSF since single amino acid substitutions can potentially alter protein folding and stability.^27^ As shown in Table 1, the *T*_*m,app*_ of all *Bt*AS variants ranged from 59.79 °C (G374L) to 61.68 °C (G374Q), while WT exhibited a *T*_*m,app*_ of 59.98 °C. Most substitutions maintained thermal stability similar to the WT, with the majority of variants displaying *T*_*m,app*_ values within approximately 1 °C of the WT. This suggests that these variants likely fold properly and possess stability profiles comparable to the original enzyme.

**Table 1:** Specific Activity and Melting Temperature of *Bt*AS WT and G374 Variants

Enzyme	Specific activity (U/mg)	Apparent melting temperature (°C)
*Bt*AS-G374A	2.37 ± 0.05^D,^ ^a^	61.22 ± 0.02
*Bt*AS-G374R	0.64 ± 0.00^J^	61.00 ± 0.01
*Bt*AS-G374N	1.62 ± 0.04^GH^	60.91 ± 0.03
*Bt*AS-G374D	1.24 ± 0.02^I^	60.62 ± 0.01
*Bt*AS-G374C	2.05 ± 0.03^EF^	61.06 ± 0.01
*Bt*AS-G374Q	1.74 ± 0.02^G^	61.68 ± 0.07
*Bt*AS-G374E	1.62 ± 0.02^GH^	61.43 ± 0.01
*Bt*AS-G374H	1.44 ± 0.02^HI^	61.04 ± 0.01
*Bt*AS-G374I	1.29 ± 0.02^I^	60.21 ± 0.01
*Bt*AS-G374L	2.03 ± 0.02^F^	59.79 ± 0.01
*Bt*AS-G374K	1.63 ± 0.00^GH^	61.52 ± 0.01
*Bt*AS-G374M	2.79 ± 0.03^C^	60.89 ± 0.00
*Bt*AS-G374F	2.95 ± 0.11^C^	60.66 ± 0.03
*Bt*AS-G374P	0.80 ± 0.01^J^	60.09 ± 0.02
*Bt*AS-G374S	2.38 ± 0.03^D^	61.09 ± 0.01
*Bt*AS-G374T	4.73 ± 0.07^B^	61.25 ± 0.01
*Bt*AS-G374W	11.48 ± 0.40^A^	60.76 ± 0.02
*Bt*AS-G374Y	2.11 ± 0.02^EF^	61.06 ± 0.02
*Bt*AS-G374V	2.25 ± 0.02^DE^	60.63 ± 0.04
*Bt*AS-WT	1.52 ± 0.03^H^	59.98 ± 0.02

a Values are means ± standard deviations. Different superscript letters indicate statistically significant differences in specific activity (*p* < 0.05, one-way ANOVA followed by Tukey’s post hoc test).

### 3.2 Product Profiles at Different Substrate Concentrations

*Bt*AS G374 variants were analyzed for their product distribution patterns under low (0.1 M) and high (2.0 M) sucrose concentrations, as substrate concentration is known to influence the proportion of isomerization versus polymerization reactions.^28^ At a high sucrose concentration (2.0 M), all *Bt*AS G374 variants exhibited significantly increased isomerization and decreased polymerization compared to reactions at low sucrose concentration (0.1 M) (Table 2). However, several variants demonstrated incomplete substrate utilization at 2.0 M sucrose, with residual sucrose levels ranging from 0.40% to 20.22%. Among these, G374K, G374E, and G374D showed particularly high residual sucrose (20.22%, 16.43%, and 14.30%, respectively), indicating reduced catalytic efficiency. In contrast, G374H and G374R displayed exceptional isomerization proportion (88.64% and 88.09%, respectively), representing approximately twice that of the WT (43.02%). Additionally, G374T exhibited a remarkable concentration-dependent shift, maintaining nearly complete substrate utilization while showing a 3.5-fold increase in isomerization proportion (from 21.01% to 72.53%). These findings suggest that specific amino acid substitutions at the +2 subsite can significantly affect both substrate utilization and product distribution patterns under varying sucrose concentrations.

**Table 2:** Product Distribution of *Bt*AS G374 Variants Using 0.1 or 2.0 M Sucrose as substrate^a^

		Proportions of products using glucose from sucrose (%)			
Enzyme	Sucrose concentration (M)	Residual sucrose	Hydrolysis	Polymerization	Isomerization
*Bt*AS-G374A	0.1	0.00 ± 0.00	18.13 ± 0.11^GHI^	44.87 ± 1.14^E^	37.01 ±1.25^BC^
	2.0	0.00 ± 0.00^i,1^	0.64 ± 0.10^cd^	18.48 ± 0.39^hij^	80.89 ± 0.50^de^
*Bt*AS-G374R	0.1	0.00 ± 0.00	4.64 ± 0.09^K^	54.31 ± 1.24^CDE^	41.05 ± 1.34^B^
	2.0	8.77 ± 0.61^f^	2.14 ± 0.24^b^	9.77 ± 0.38^m^	88.09 ±0.14^a^
*Bt*AS-G374N	0.1	0.00 ± 0.00	17.41 ± 0.03 ^HI^	51.73 ± 3.68^DE^	30.86 ± 3.72^BCDEF^
	2.0	1.29 ± 0.24^i^	0.57 ± 0.05^cdef^	15.97 ± 0.74^kl^	83.46 ± 0.69^bc^
*Bt*AS-G374D	0.1	0.00 ± 0.00	16.36 ± 0.01^HI^	67.87 ± 1.61^A^	15.77 ± 1.60^GHI^
	2.0	14.3 ± 0.45^c^	0.27 ± 0.01^efg^	22.30 ±0.26^ef^	77.43 ± 0.25^gh^
*Bt*AS-G374C	0.1	0.00 ± 0.00	18.66 ± 0.05^FGH^	61.38 ± 0.15^ABC^	19.96 ±0.20^FGH^
	2.0	7.07 ± 1.09^g^	0.29 ± 0.01^efg^	20.23 ± 0.07^gh^	79.48 ± 0.08^ef^
*Bt*AS-G374Q	0.1	0.00 ± 0.00	18.65 ± 0.05^FGH^	58.24 ± 0.19^BCD^	23.11 ± 0.14^DEFGH^
	2.0	9.63 ± 0.37^ef^	0.25 ± 0.02^fg^	17.27 ± 0.19^ijk^	82.48 ± 0.16^cd^
*Bt*AS-G374E	0.1	0.00 ± 0.00	19.84 ± 0.10^EFG^	68.16 ± 0.30^A^	12.01 ± 0.20^HI^
	2.0	16.43 ± 0.49^b^	0.25 ± 0.08^fg^	19.04 ± 0.05^hi^	80.71 ± 0.04^de^
*Bt*AS-G374H	0.1	0.00 ± 0.00	9.92 ± 0.02^J^	31.82 ± 0.09^F^	58.25 ± 0.11^A^
	2.0	0.40 ± 0.03^i^	2.65 ± 0.20^a^	8.71 ± 0.72^m^	88.64 ± 0.51^a^
*Bt*AS-G374I	0.1	0.00 ± 0.00	34.54 ± 0.11^B^	50.10 ± 0.16^DE^	15.37 ± 0.27^GHI^
	2.0	12.31 ± 0.46^d^	0.79 ± 0.17^c^	14.67 ± 0.40^l^	84.54 ± 0.57^b^
*Bt*AS-G374L	0.1	0.00 ± 0.00	23.77 ± 1.09^D^	49.37 ± 7.56^DE^	26.86 ±8.65^CDEFG^
	2.0	6.15 ± 0.15^g^	0.41 ± 0.12^def^	16.04 ± 0.01^kl^	83.55 ± 0.11^bc^
*Bt*AS-G374K	0.1	0.00 ± 0.00	16.31 ± 1.70^HI^	50.80 ± 2.39^DE^	32.89 ±4.09^BCD^
	2.0	20.22±1.61^i^	0.36 ± 0.04^def^	14.88 ± 0.26^l^	84.76±0.22^b^
*Bt*AS-G374M	0.1	0.00 ± 0.00	20.76 ± 2.34^EF^	46.10 ± 8.42^E^	33.13±10.76^BCD^
	2.0	6.73 ± 0.66^g^	0.47 ± 0.09^def^	16.99 ± 0.57^jk^	82.54±0.48^cd^
*Bt*AS-G374F	0.1	0.00 ± 0.00	23.90 ± 0.00^D^	51.39 ± 0.08^DE^	24.71±0.08^DEFG^
	2.0	1.58 ± 0.40^hi^	0.30 ± 0.03^efg^	23.47 ± 0.29^de^	76.23±0.26^hi^
*Bt*AS-G374P	0.1	0.00 ± 0.00	16.28 ± 0.16^I^	56.22 ±0.31^BCD^	27.50±0.14^CDEF^
	2.0	0.00 ± 0.00^i^	0.47 ± 0.05^def^	25.05 ± 0.45^d^	74.47±0.4^i^
*Bt*AS-G374S	0.1	0.00 ± 0.00	17.16 ± 0.34^HI^	50.62 ± 0.34^DE^	32.21±0.68^BCDE^
	2.0	0.00 ± 0.00^i^	0.33 ± 0.04^def^	20.95 ± 0.22^fg^	78.72±0.18^fg^
*Bt*AS-G374T	0.1	0.00 ± 0.00	27.36 ± 0.13^C^	51.63 ± 1.02^DE^	21.01±1.15^EFGH^
	2.0	0.40 ± 0.01^i^	0.43 ± 0.01^def^	27.04 ± 0.11^c^	72.53±0.10^j^
*Bt*AS-G374W	0.1	0.00 ± 0.00	38.04 ± 0.41^A^	29.29 ± 0.50^F^	32.67±0.91^BCDE^
	2.0	0.00 ± 0.00^i^	0.58 ± 0.04^cde^	23.41 ± 0.16^de^	76.00±0.20^hi^
*Bt*AS-G374Y	0.1	0.00 ± 0.00	21.07 ± 0.09^E^	56.81 ± 0.31^BCD^	22.11±0.21^DEFGH^
	2.0	2.96 ± 0.40^h^	0.32 ± 0.03^ef^	23.8 ± 0.44^de^	75.88±0.41^hi^
*Bt*AS-G374V	0.1	0.00 ± 0.00	28.74 ± 0.31^C^	64.11 ± 0.70^AB^	7.15±1.01^I^
	2.0	11.10 ± 0.12^DE^	0.30 ± 0.01^EFG^	29.26 ± 0.25^b^	70.43±0.26^k^
*Bt*AS-WT	0.1	0.00 ± 0.00	11.53 ± 0.01^J^	63.99 ± 2.63^AB^	24.48±2.63^DEFG^
	2.0	0.00± 0.00^I^	0.00 ± 0.00^G^	56.98 ± 2.17^a^	43.02±2.17^l^

a Reactions were performed in 50 mM sodium acetate buffer (pH 6.0) at 50 °C for 24 h. Values are means ± standard deviations. Different superscript letters (uppercase (A: 0.1 M sucrose; lowercase: 2.0 M sucrose) within the same column indicate statistically significant differences (*p* < 0.05, one-way ANOVA followed by Tukey’s post hoc test). Uppercase letter (A-H) represent 0.1M sucrose, and lowercase letter (a-k) represent 2.0M sucrose.

Production profile analysis revealed characteristic differences in catalytic activity among G374 variants compared to the WT (Figure 1). At low sucrose concentration (0.1 M), G374H showed the most pronounced increase in turanose production (Figure 1A), while at high concentration (2.0 M), all variants exhibited dramatically enhanced product formation (Figure 1B). A previous study reported that the G374S achieved a high turanose production yield of 65.0% in 2 M sucrose while the WT produced only 25.20%.^23^ However, G374S demonstrated only slightly improved turanose yield of 24.04% compared to WT (15.00% at 0.1 M sucrose. In contrast, G374H exhibited notably elevated turanose yield of 55.63% under the same low concentration conditions. This enhanced performance suggests that the histidine substitution at the +2 subsite may alter the microenvironment of the catalytic pocket, thereby affecting substrate binding and product specificity. Previous studies have shown that the +1/+2 subsites of ASase are particularly important for determining whether turanose or trehalulose is formed during isomerization.^12,14,29^ The histidine residue in G374H can act as both hydrogen bond donor and acceptor,^30^ which may facilitate interactions with the fructose moiety. These properties might enhance fructose stabilization in an orientation that favors α-(1→3) linkage formation (turanose) over α-(1→1) linkage formation (trehalulose), explaining the exceptional turanose selectivity of G374H across different sucrose concentrations. Conversely, G374E and G374D showed the lowest turanose production even at low sucrose concentrations and showed significantly reduced catalytic efficiency at high sucrose concentrations, failing to fully utilize sucrose. This phenomenon could be attributed to the negative charges of glutamate and aspartate creating unfavorable electrostatic repulsions with the hydroxyl groups of fructose, potentially disrupting optimal substrate binding orientation.^31^ Interestingly, G374T and G374V both showed increased trehalulose production, unlike other variants that primarily enhanced turanose formation. However, G374V failed to completely utilize sucrose at 2.0 M concentration, while G374T maintained efficient sucrose conversion (Figure 1B). These results are consistent with previous studies showing that +2 subsite mutations alter product selectivity without compromising catalytic activity.^32,33^ *Dg*AS produces both trehalulose and turanose, with a higher ratio of trehalulose production compared to *Np*AS.^14,34^ Crystal structure analysis of *Dg*AS showed that specific residues at +1/+2 subsites create a hydrophobic pocket that orients fructose to favor α-(1→1) linkage formation. Therefore, the +2 subsite of *Bt*AS G374 might play a key role in determining isomer selectivity through active site modifications. The achieved isomer selectivity of 88.64% by G374H represents substantial improvement from wild-type (∼43% and is among the highest reported for direct ASase-catalyzed reactions. This level of selectivity is suitable for functional food or prebiotic applications, where structurally related isomer mixtures (turanose and trehalulose) may provide synergistic effects. For high-purity applications, downstream processing would be required, similar to standard practice in amylosucrase-based bioprocesses.

**Figure 1:**
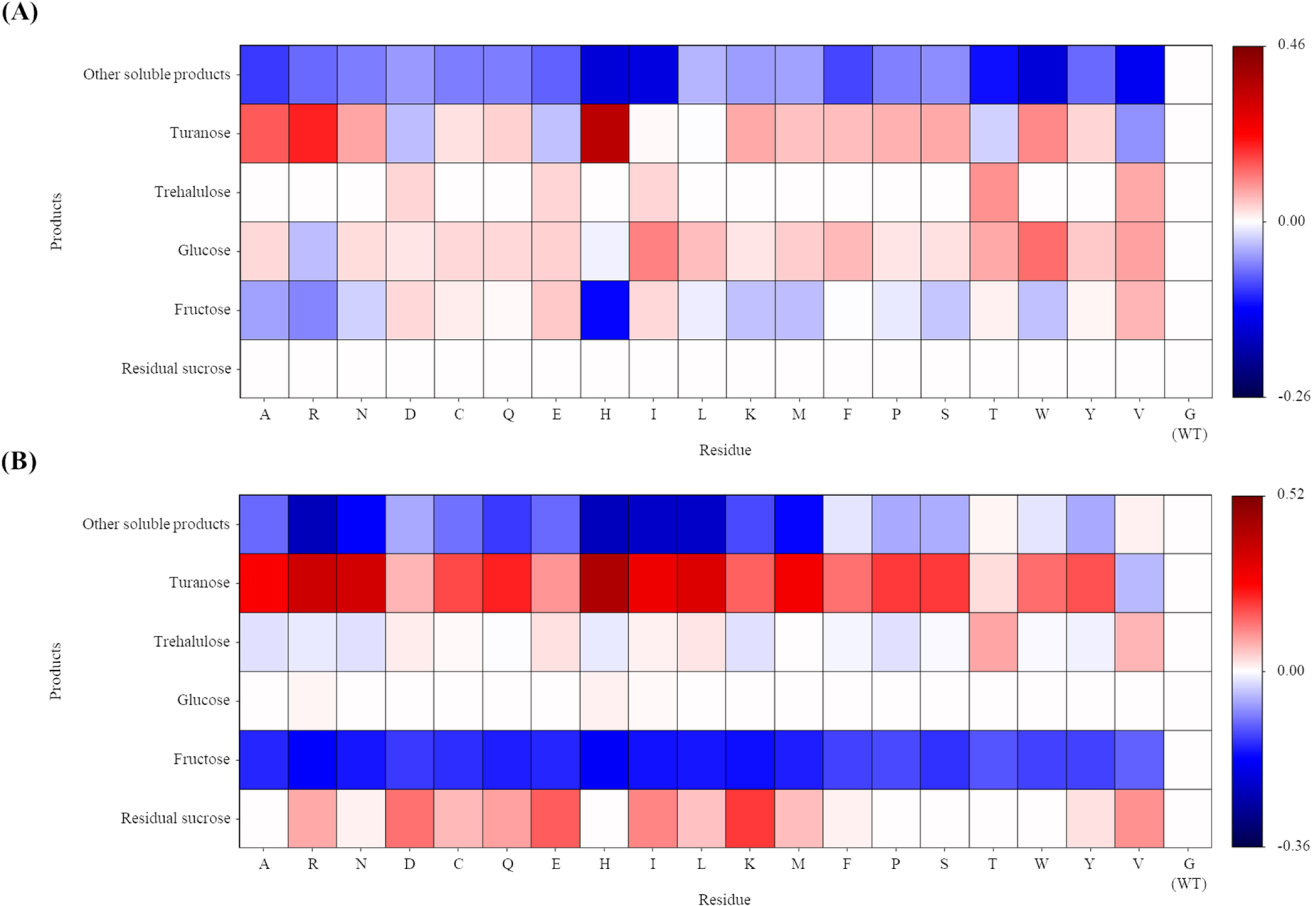
Heatmaps showing reaction product profiles of *Bt*AS G374 variants relative to wild-type enzyme. Reactions were performed in 50 mM sodium acetate buffer (pH 6.0) at 50 °C for 24 h using (A) 0.1 M or (B) 2.0 M sucrose as substrate. Color scale indicates fold-change relative to WT (red, higher than WT; blue, lower than WT; white, equal to WT).

### 3.3 Enzymatic Characterization of *Bt*AS G374H and G374T Variants

G374H and G374T were selected for detailed enzymatic characterization as they demonstrated both complete sucrose utilization across all tested concentrations and the highest production yields of turanose and trehalulose, respectively. The effects of temperature and pH on enzyme activity were investigated for both G374H and G374T variants. The optimal reaction temperature for all three enzymes was 50 °C (Figure S3), and the pH profiles of both variants closely resembled that of the WT, with optimal activity at pH 6.0 in sodium acetate buffer (Figure S4). Previous studies showed that G374S also maintained optimal temperature and pH profiles consistent with the WT.^15,23^ These results indicate that mutations at residue 374 do not significantly alter the temperature or pH dependency of *Bt*AS activity.

Product formation was monitored over time using 2.0 M sucrose (684.6 g/L) under these optimal reaction conditions (Figure 2). Both G374H and G374T variants significantly altered their product formation profiles compared to the WT. Specifically, G374H exhibited remarkably enhanced turanose production, reaching approximately 615 g/L within 12 h and maintaining this level throughout the 72 h reaction period, representing more than a 2-fold increase compared to the WT (approximately 230 g/L). Conversely, G374T demonstrated superior trehalulose production, reaching 80 g/L after 36 h compared to only approximately 15 g/L for the WT. Additionally, G374H showed reduced trehalulose production compared to the WT, further confirming its selectivity toward turanose formation. Analysis of the residual sucrose concentration profiles revealed different substrate utilization patterns among the enzymes. G374H consumed sucrose more rapidly than the WT, while G374T showed slightly slower substrate consumption compared to the WT (Figure 2C). All three enzymes completely utilized the sucrose within 36 h. These results demonstrate that single amino acid substitutions at position 374 can dramatically alter both the substrate utilization rates and product selectivity in *Bt*AS.

**Figure 2:**
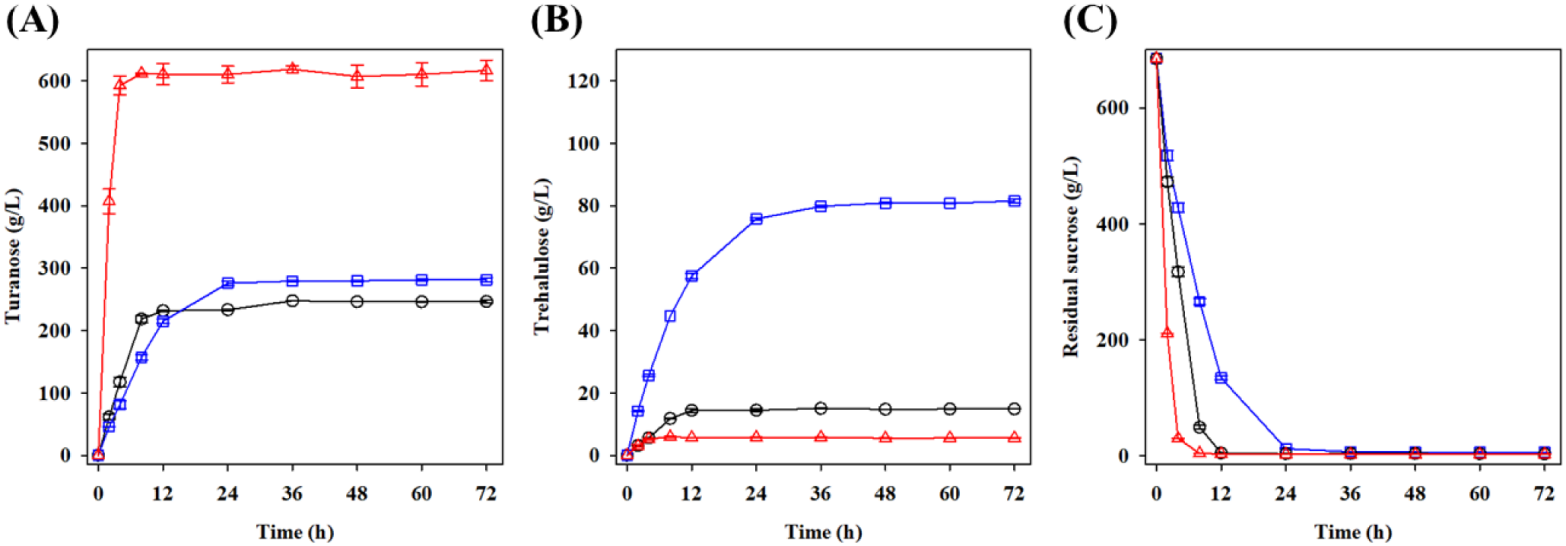
Time-course analysis of turanose and trehalulose production by *Bt*AS WT, G374H, and G374T variants. Reactions were performed with 2.0 M sucrose as substrate in 50 mM sodium acetate buffer (pH 6.0) at 50 °C. (A) Turanose concentration; (B) trehalulose concentration; (C) residual sucrose concentration. Symbols: black circle, *Bt*AS-WT; red triangle, *Bt*AS-G374H; blue square, *Bt*AS-G374T.

### 3.4 Kinetic Analysis of *Bt*AS G374H and G374T Variants

Previous studies reported that ASase catalyzes isomerization reactions when fructose serves as an acceptor molecule, whether endogenous (from sucrose hydrolysis) or exogenous (added externally).^35^ Kinetic studies were performed to assess the catalytic efficiency of *Bt*AS variants toward fructose-dependent isomerization (Table 3). Sucrose concentration was maintained constant at 100 mM while fructose concentration was varied from 18 to 100 mM. The *Bt*AS variants exhibited significantly different kinetic parameters compared to the WT. For turanose production, G374H demonstrated a remarkable 9.1-fold increase in catalytic efficiency (*k*_*cat*_ */K*_*m*_ = 4.89 ± 0.41 mM^–1^·min^–1^) compared to the WT (*k*_*cat*_ */K*_*m*_ = 0.54 ± 0.02 mM^–1^·min^–1^). This improvement was primarily due to a 6.3-fold enhancement in turnover number (*k*_*cat*_ = 275.03 ± 0.02 min^–1^ compared to 43.73 ± 2.94 min^–1^) and a moderate decrease in *K*_*m*_ (56.20 ± 3.95 mM compared to 77.06 ± 5.65 mM). G374T also showed improved catalytic efficiency for turanose formation (*k*_*cat*_ */K*_*m*_ = 1.54 ± 0.01 mM^–1^·min^–1^), with reduced *K*_*m*_ (36.03 ± 3.95 mM) indicating enhanced acceptor affinity. For trehalulose production, G374T exhibited substantially higher catalytic parameters compared to the WT, with a 7.8-fold increase in catalytic efficiency (*k*_*cat*_ */K*_*m*_ = 1.17 ± 0.01 mM^–1^·min^–1^ compared to 0.15 ± 0.00 mM^–1^·min^–1^). This improvement resulted from a significant increase in *k*_*cat*_ (39.61 ± 2.38 min^–1^ compared to 1.61 ± 0.01 min^–1^). Kinetic parameters for trehalulose production by G374H could not be determined due to insufficient trehalulose formation under the experimental conditions. The substantial improvements in *k*_*cat*_ suggest that these substitutions at the +2 subsite facilitate faster catalytic turnover, potentially by optimizing the chemical step of glycosidic bond formation or improving product release kinetics. More importantly, these kinetic changes were accompanied by distinct product selectivity patterns: G374H appeared to favor α-1,3 linkage formation (turanose) while showing reduced α-1,1 linkage formation (trehalulose), whereas G374T predominantly enhanced α-1,1 linkage formation. This selective enhancement of specific reaction pathways suggests that the +2 subsite plays a key role in determining fructose positioning within the active site, thereby affecting both catalytic efficiency and product specificity.

**Table 3:** Kinetic Parameters of *Bt*AS Variants for Isomerization Reactions Using Fructose as Acceptor.^a^

	Turanose production			Trehalulose production	
Apparent kinetic constants	*Bt*AS-WT	*Bt*AS-G374H	*Bt*AS-G374T	*Bt*AS-WT	*Bt*AS-G374T
*V*_max_ (μmol/min·mg)	0.61 ± 0.03^A,^ ^b^	3.98 ± 0.14^C^	0.80 ± 0.03^B^	0.02 ± 0.00	0.57 ± 0.02*,^c^
*K*_*m*_ (mM)	77.06 ± 5.64^C^	56.20 ± 3.95^B^	36.03 ± 3.19^A^	10.78 ± 0.47	34.02 ± 2.87*
*k*_*cat*_ (min^–1^)	43.73 ± 2.94^A^	275.03 ± 4.84^C^	55.47 ± 3.54^B^	1.61 ± 0.01	39.61 ± 2.38*
*k*_*cat*_ */K*_*m*_ (mM^–1^·min^–1^)^d^	0.54 ± 0.02^A^	4.89 ± 0.41^C^	1.54 ± 0.01^B^	0.15 ± 0.00	1.17 ± 0.01*

a Reactions were performed at 50 °C in 50 mM sodium acetate buffer (pH 6.0) with 100 mM sucrose and varying fructose concentrations (18–100 mM).

b Values are means ± standard deviations. Different superscript letters (A-D) indicate statistically significant differences among turanose production parameters (*p* < 0.05, one-way ANOVA).

c Asterisks (*) indicate significant differences between wild-type and G374T for trehalulose production (*p* < 0.05, Student’s *t* test).

d *k*_*cat*_ */K*_*m*_(mM^–1^ · min^–1^) = catalytic efficiency.

### 3.5 Structural Analysis and Molecular Dynamics Simulation of *Bt*AS Variants

Three-dimensional models of WT, G374H, and G374T were generated using the AlphaFold3 to understand the structural basis of altered product specificity in the *Bt*AS variants. All predicted structures exhibited high pLDDT scores (>96), suggesting high confidence and reliability of the models^36^ (Figure S5). Three distinct molecular states were modeled for each enzyme: enzyme-sucrose complex, glycosyl-enzyme intermediate with docked fructose, and enzyme–product complexes with either turanose or trehalulose, representing the 3 key stages of a catalytic cycle^14,32,37^ (Figure S6). Analysis of the glycosyl-enzyme intermediate complexes revealed distinct fructose positioning patterns among the variants that explain their different product specificities (Figure 3). In the G374H, that demonstrated the greatest turanose production, fructose was optimally oriented for α-(1→3) glycosidic bond formation (Figure 3B). The distance between the C3-OH group of fructose and the C1 position of glucose was 2.7 Å, which was significantly shorter than that observed in the WT (Figure 3A). In contrast, G374T showed a different fructose binding pattern that favored trehalulose formation. The C1-OH groups of glucose and fructose were positioned within 3.2 Å of each other, substantially closer than the 7.0 Å distance observed in the WT (Figure 3A,C). Analysis of substrate channel and product tunnel architecture across the catalytic cycle further revealed that G374H exhibits enhanced channel flexibility while G374T displays a more structured, preorganized binding pocket (Figure S7).

**Figure 3:**
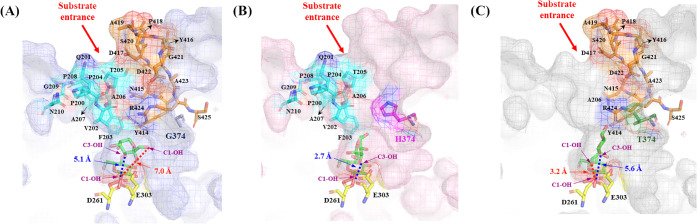
Three-dimensional structures of *Bt*AS variants in the glycosyl-enzyme intermediate state with docked fructose, showing distinct fructose positioning patterns. (A) *Bt*AS-WT; (B) *Bt*AS-G374H; (C) *Bt*AS-G374T. Distances (Å) indicate the proximity between the nucleophilic oxygen of fructose (O3 or O1) and the anomeric carbon (C1) of the glycosyl-intermediate.

Molecular dynamics (MD) simulations were performed to investigate how substitution at residue 374 influences protein flexibility and the local interaction network around the +2 subsite, thereby affecting product specificities. Root mean square deviation (RMSD) analysis across all simulations showed stable trajectories beyond 100 ns, confirming reliable conformational sampling throughout the simulation period. In the enzyme-only state, all variants exhibited lower RMSD values compared to the WT, indicating enhanced structural stability upon mutation, which is consistent with their maintained thermal stability profiles^38^ (Figure S8A). When complexed with their respective products, all enzymes maintained similar RMSD values, suggesting stable product-bound conformations (Figure S8B–D). Notably, in the glycosyl-enzyme intermediate state, G374H displayed a higher RMSD value than other enzymes after 30 ns (Figure S8B). This pattern suggests increased conformational flexibility during the catalytic process.

Single amino acid substitutions in the ASase can significantly modulate loop dynamics, thereby affecting both reaction efficiency and the relative proportions of reaction products.^29,39^ Root mean square fluctuation (RMSF) analysis, which quantifies the flexibility of each amino acid residue during MD simulation,^40^ revealed that G374H and G374T exhibited altered flexibility patterns at specific residues compared to the WT (Figure 4, Figure S9 and Figure S10). Analysis of specific loop regions revealed distinct patterns. Loop 3 constitutes a key structural element in ASase that defines the active site configuration and oligosaccharide binding subsites.^12,29,41^ Changes in loop flexibility and amino acid composition within this region directly affect acceptor recognition, substrate affinity, and transglycosylation efficiency.^29,42^ In loop 3 (residues 200–210), G374H exhibited increased flexibility in the glycosyl-enzyme intermediate with fructose state compared to the WT (Figure 4A). As illustrated in Figure 3B, Figure S8 and Figure S9, this increased flexibility (0.5–0.8 Å RMSF increase) allows the enzyme to accommodate the acceptor more effectively. This structural adaptability results in a precise positioning of the fructose C3-OH at 2.7 Å from the glycosyl-intermediate, significantly reducing the catalytic distance (measured between the fructose nucleophilic oxygen and the glucose C1 carbon) compared to the WT (5.1 Å, Figure 3A). Conversely, G374T showed decreased flexibility in the same region during the intermediate state compared to G374H (Figure 4B). The increased flexibility in loop 3 of G374H facilitates dynamic acceptor recognition and optimal fructose positioning. Supporting this mechanism, Ile221 substitution in *Dg*AS altered loop 3 flexibility and enhanced acceptor accessibility, while Arg226 in *Np*AS loop 3 stabilizes fructose for turanose synthesis.^17,29^ Our kinetic analysis supports these flexibility effects, as G374H showed improved acceptor binding affinity (lower *K*_*m*_), while G374T demonstrated enhanced catalytic turnover that favors trehalulose formation. The loop containing residue 374 (residues 370–390) displayed the most pronounced flexibility alterations. G374H showed dramatically decreased flexibility in the intermediate state, which increased upon turanose complex formation. G374T exhibited moderate flexibility increases in the same region with less dramatic changes than G374H. The enhanced flexibility in G374H enables the observed optimal fructose C3-OH positioning at 2.7 Å distance, facilitating turanose formation. In contrast, the moderate flexibility changes in G374T support the 3.2 Å C1-OH positioning required for trehalulose synthesis (Figure 3). This distance is consistent with the optimal range (3.0–3.3 Å) previously reported for effective nucleophilic attack during transglycosylation.^20,36,43^ Loop 7 (residues 414–425) exhibited distinct flexibility patterns between the variants. G374T showed significantly increased flexibility in this region, particularly in the trehalulose complex state compared to the WT. In contrast, G374H displayed relatively minor changes in loop 7 flexibility across all states. This enhanced flexibility in loop 7 of G374T correlates directly with the observed 7.8-fold increase in catalytic efficiency for trehalulose production. To quantitatively confirm that these dynamics translate to efficient product dissociation,^44^ we performed tunnel analysis using CAVER 3.0.3. Adopting the quantitative approach recently established for discriminating transglycosylated product-release pathways,^19^ we evaluated the exit tunnels of the variants. As summarized in Figure S11, the G374T variant possessed a significantly optimized exit pathway compared to the WT. The average bottleneck radius (Avg_BR) of G374T was slightly larger (2.604 Å), and its average length (Avg_L) was substantially shorter (1.996 Å) than those of the WT (Avg_BR = 2.565 Å; Avg_L = 2.452 Å). Furthermore, the near-perfect linearity of the G374T tunnel (Avg_C = 1.004) suggests minimal physical hindrance during trehalulose dissociation. These quantitative metrics strongly substantiate that the G374T mutation at the subsite +2 promotes catalytic turnover by engineering a more efficient product exit tunnel, effectively discriminating the transglycosylated product-release pathway. Collectively, these findings demonstrate that single amino acid substitutions at the +2 subsite in ASase can induce coordinated structural changes across multiple active site loops, ultimately determining product specificity through coordinated alterations in substrate recognition, catalytic efficiency, and product release.

**Figure 4:**
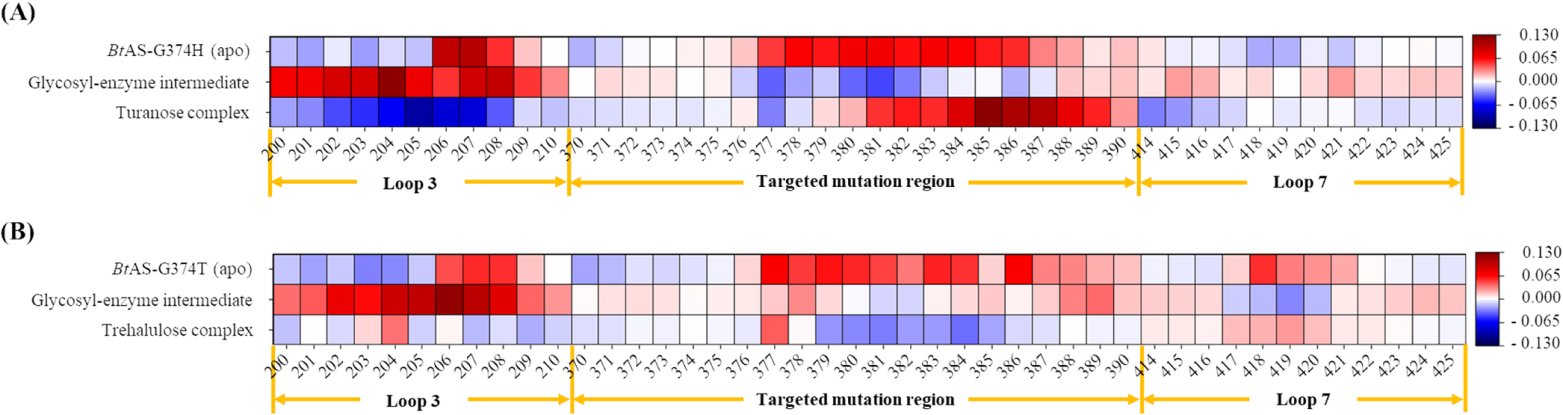
Comparative heatmaps of residue-level flexibility for *Bt*AS variants (G374H and G374T) relative to the WT. Values represent the fold-change in RMSF for each variant across different catalytic states. (A) Flexibility profile for the *Bt*AS-G374H variant; (B) Flexibility profile for the *Bt*AS-G374T variant. The color scale indicates flexibility changes compared to the WT: red, increased flexibility; blue, decreased flexibility; white, no change.

In conclusion, this study demonstrates that single amino acid substitutions at the +2 subsite (Gly374) of *Bt*AS can significantly enhance catalytic efficiency and alter product selectivity. Among 19 variants, G374H and G374T showed the most pronounced improvements in turanose and trehalulose production, respectively. G374H exhibited a 9.1-fold increase in catalytic efficiency (*k*_*cat*_/*K*_*m*_) for turanose formation and achieved 88.64% product selectivity (relative to total isomers) under high substrate concentrations, resulting in significantly enhanced product yield. G374T demonstrated a 7.8-fold improvement in catalytic efficiency for trehalulose production. Molecular dynamics simulations revealed that these variants adopt distinct catalytic strategies. G374H utilizes enhanced conformational flexibility for optimal substrate positioning, while G374T employs preorganized binding and efficient product release mechanisms. The structural analyses showed that substitutions at the +2 subsite induce coordinated changes across multiple active site loops, simultaneously affecting substrate recognition, catalytic efficiency, and product release. These findings demonstrate that the +2 subsite serves as a critical control point for enzyme function and provide valuable insights for engineering ASase variants with enhanced selectivity and yield for functional oligosaccharide production. Building upon the structural blueprint established at the +2 subsite, future studies will explore multisite mutagenesis to harness potential synergistic effects for further enhancing ASase catalytic efficiency.

## Supplementary Material



## Notes

Table S1: oligonucleotide sequences for site-saturation mutagenesis of *Bt*AS at position G374; **Table S2**: strains, plasmids, and primers used in this study; **Figure S1**: proposed catalytic mechanism for sucrose isomerization by amylosucrase. The schematic illustrates the initial formation of the glycosyl-enzyme intermediate from sucrose and the subsequent nucleophilic attack by the fructose acceptor. Specifically, the reaction via the C1-OH group (highlighted in red) leads to the synthesis of trehalulose, whereas the reaction via the C3-OH group (highlighted in blue) leads to the formation of turanose; **Figure S2**: SDS-PAGE analysis of *Bt*AS variants with mutations at residue 374 expressed in *E. coli* BL21­(DE3). M: Marker; 1–2: *Bt*AS wild-type (cell extract and purified enzyme); 3–4: *Bt*AS_G374H (cell extract and purified enzyme); 5–6: *Bt*AS_G374C (cell extract and purified enzyme); 7–8: *Bt*AS_G374Q (cell extract and purified enzyme); 9–10: *Bt*AS_G374T (cell extract and purified enzyme); 11–12: *Bt*AS_G374E (cell extract and purified enzyme); 13–14: *Bt*AS_G374D (cell extract and purified enzyme); 15–16: *Bt*AS_G374L (cell extract and purified enzyme); 17–18: *Bt*AS_G374 M (cell extract and purified enzyme); 19–20: *Bt*AS_G374F (cell extract and purified enzyme); 21–22: *Bt*AS_G374A (cell extract and purified enzyme); 23–24: *Bt*AS_G374R (cell extract and purified enzyme); 25–26: *Bt*AS_G374 V (cell extract and purified enzyme); 27–28: *Bt*AS_G374I (cell extract and purified enzyme); 29–30: *Bt*AS_G374W (cell extract and purified enzyme); 31–32: *Bt*AS_G374 K (cell extract and purified enzyme); 33–34: *Bt*AS_G374P (cell extract and purified enzyme); 35–36: *Bt*AS_G374Y (cell extract and purified enzyme); 37–38: *Bt*AS_G374N (cell extract and purified enzyme); 39–40: *Bt*AS_G374G (cell extract and purified enzyme); 41–42: *Bt*AS_G374S (cell extract and purified enzyme); **Figure S3**: effect of temperature on enzyme activity of *Bt*AS WT, G374H, and G374T variants. Reactions were performed at various temperatures for 30 min using 0.1 M sucrose as substrate in 50 mM sodium acetate buffer (pH 6.0). (A) *Bt*AS-WT; (B) *Bt*AS-G374H; (C) *Bt*AS-G374T; **Figure S4**: effect of pH on enzyme activity of *Bt*AS WT, G374H, and G374T variants. Reactions were performed at 50 °C for 30 min using 0.1 M sucrose as substrate. (A) BtAS-WT; (B) *Bt*AS-G374H; (C) *Bt*AS-G374T. Symbols indicate different buffer systems: black triangle, 50 mM sodium acetate; black circle, 50 mM sodium phosphate; black rhombus, 50 mM Tris-HCl; black square, 50 mM glycine-NaOH; **Figure S5**: per-Residue confidence scores (pLDDT) for AlphaFold3-predicted structures of *Bt*AS WT, G374H, and G374T variants in different molecular states. The pLDDT scores indicate structural confidence, with higher values representing more reliable predictions. “-” indicates that the structure was not modeled for the corresponding state; **Figure S6**: three-dimensional structures of *Bt*AS variants showing different catalytic states. (A–C) enzyme-sucrose complexes: (A) *Bt*AS-WT; (B) *Bt*AS-G374H; (C) *Bt*AS-G374T. (a–c) Glycosyl-enzyme intermediates with docked fructose: (a) *Bt*AS-WT; (b) *Bt*AS-G374H; (c) *Bt*AS-G374T. (i–iii) Product complexes: (i) *Bt*AS-WT with turanose; (ii) *Bt*AS-G374H with turanose; (iii) *Bt*AS-G374T with trehalulose; **Figure S7**: substrate channel architecture during *Bt*AS catalysis. Channel/tunnel visualization for (A) WT, (B) G374H, (C) G374T. Upper: sucrose complex; middle: intermediate with fructose; lower: product complex. Mesh shows accessible volume. Yellow: catalytic residues; blue: channel residues; green/magenta: position 374. G374H exhibits flexible channel for turanose formation; G374T shows structured pocket for trehalulose production; **Figure S8**: Root Mean Square Deviation (RMSD) analysis of *Bt*AS WT, G374H, and G374T variants over 100 ns of molecular dynamics simulations. (A) Enzyme-only (apo) state; (B) glycosyl-enzyme intermediate state with docked fructose; (C) enzyme complexes with turanose; (D) enzyme complexes with trehalulose; **Figure S9**: root mean square fluctuation (RMSF) analysis of *Bt*AS WT and G374H variant over 100 ns of molecular dynamics simulations. (A) *Bt*AS-WT; (B) *Bt*AS-G374H. Key structural regions (loop 3 and targeted mutation zone) are highlighted; **Figure S10**: root mean square fluctuation (RMSF) analysis of *Bt*AS WT and G374T variant over 100 ns of molecular dynamics simulations. (A) *Bt*AS-WT; (B) *Bt*AS-G374T. Key structural regions (loop 7 and targeted mutation zone) are highlighted; **Figure S11**: quantitative analysis of product exit tunnels in WT and G374 variants. Detailed tunnel properties calculated using CAVER 3.0.3 are summarized in the table. The Average Bottleneck Radius (Avg_BR), Average Length (Avg_L), and Average Curvature (Avg_C) represent the physical dimensions and efficiency of the pathways for turanose and trehalulose dissociation (PDF)

## Abbreviations

*Bt*AS: ASase from *Bifidobacterium thermophilum*
*Np*AS: ASase from *Neisseria polysaccharea*
HPLC-ELSD: High-performance liquid chromatography coupled with evaporative light scattering detection
HPAEC-PAD: High-performance anion-exchange chromatography with pulsed amperometric detection
MD: Molecular dynamics
CGenFF: CHARMM General Force Field
LINCS: Linear Constraint Solver
RMSD: Root mean square deviation
RMSF: Root-mean-square fluctuation
