# Supplementary material for: Modulation of Fructose Transfer Process for Promoting Apparent Isomerization Activity of Amylosucrase from Bifidobacterium thermophilum
Source: J Agric Food Chem. 2026 Apr 6;74(14):11714–24. doi: 10.1021/acs.jafc.6c02580 (PMC13088237; doi:10.1021/acs.jafc.6c02580)
Supplement: Supplementary file 1 [file jf6c02580_si_001.pdf]

# Supplementary materials

## Modulation of fructose transfer process for promoting apparent isomerization activity of amylosucrase from *Bifidobacterium thermophilum*

Yoon-Ji Jeong[a, †], Dong-Ho Seo[a, †], Sang-Ho Yoo[a,*]

**Author affiliation**

[a]Department of Food Science & Biotechnology and Carbohydrate Bioproduct Research Center, Sejong University, 209 Neungdong-ro, Gwangjin-gu, Seoul 05006, Republic of Korea

* Corresponding author:

Sang-Ho Yoo, Tel.: +82 2 3408 3221; fax: +82 2 3408 4319; e-mail: shyoo@sejong.ac.kr.

**Table S1.** Oligonucleotide sequences for site-saturation mutagenesis of BtAS at position G374.

| Mutants | Sequence of the oligonucleotides (5' → 3') |
|---|---|
| *Bt*AS-G374A (Ala) | 5' – 1100 TGCGCTGCCATGACGATATC**GCG**TGGGGTCTGGACGAG 1137 – 3' |
| *Bt*AS-G374R (Arg) | 5' – 1100 TGCGCTGCCATGACGATATC**CGG**TGGGGTCTGGACGAG 1137 – 3' |
| *Bt*AS-G374N (Asn) | 5' – 1100 TGCGCTGCCATGACGATATC**AAC**TGGGGTCTGGACGAG 1137 – 3' |
| *Bt*AS-G374D (Asp) | 5' – 1100 TGCGCTGCCATGACGATATC**GAT**TGGGGTCTGGACGAG 1137 – 3' |
| *Bt*AS-G374C (Cys) | 5' – 1100 TGCGCTGCCATGACGATATC**TGT**TGGGGTCTGGACGAG 1137 – 3' |
| *Bt*AS-G374Q (Gln) | 5' – 1100 TGCGCTGCCATGACGATATC**CAG**TGGGGTCTGGACGAG 1137 – 3' |
| *Bt*AS-G374E (Glu) | 5' – 1100 TGCGCTGCCATGACGATATC**GAG**TGGGGTCTGGACGAG 1137 – 3' |
| *Bt*AS-G374G (Gly) | 5' – 1100 TGCGCTGCCATGACGATATC**GGC**TGGGGTCTGGACGAG 1137 – 3' |
| *Bt*AS-G374H (His) | 5' – 1100 TGCGCTGCCATGACGATATC**CAT**TGGGGTCTGGACGAG 1137 – 3' |
| *Bt*AS-G374I (Ile) | 5' – 1100 TGCGCTGCCATGACGATATC**ATC**TGGGGTCTGGACGAG 1137 – 3' |
| *Bt*AS-G374L (Leu) | 5' – 1100 TGCGCTGCCATGACGATATC**TTG**TGGGGTCTGGACGAG 1137 – 3' |

| | |
|---|---|
| *Bt*AS-G374K (Lys) | 5’ – 1100 TGCGCTGCCATGACGATATC**AAG**TGGGGTCTGGACGAG 1137 – 3’ |
| *Bt*AS-G374M (Met) | 5’ – 1100 TGCGCTGCCATGACGATATC**ATG**TGGGGTCTGGACGAG 1137 – 3’ |
| *Bt*AS-G374F (Phe) | 5’ – 1100 TGCGCTGCCATGACGATATC**TTT**TGGGGTCTGGACGAG 1137 – 3’ |
| *Bt*AS-G374P (Pro) | 5’ – 1100 TGCGCTGCCATGACGATATC**CCG**TGGGGTCTGGACGAG 1137 – 3’ |
| *Bt*AS-G374S (Ser) | 5’ – 1100 TGCGCTGCCATGACGATATC**TCG**TGGGGTCTGGACGAG 1137 – 3’ |
| *Bt*AS-G374T (Thr) | 5’ – 1100 TGCGCTGCCATGACGATATC**ACG**TGGGGTCTGGACGAG 1137 – 3’ |
| *Bt*AS-G374W (Trp) | 5’ – 1100 TGCGCTGCCATGACGATATC**TGG**TGGGGTCTGGACGAG 1137 – 3’ |
| *Bt*AS-G374Y (Tyr) | 5’ – 1100 TGCGCTGCCATGACGATATC**TAT**TGGGGTCTGGACGAG 1137 – 3’ |
| *Bt*AS-G374V (Val) | 5’ - 1100 TGCGCTGCCATGACGATATC**GTC**TGGGGTCTGGACGAG 1137 – 3’ |

Bold and underlined sequences indicate the mutated codons where glycine at residue 374 of BtAS wild-type was substituted with each of the 19 amino acids.

**Table S2.** Strains, plasmids, and primers used in this study.

| Type | Name | Description / Sequence | Purpose | Reference |
|---|---|---|---|---|
| Strain | *E. coli* DH5α | High-efficiency cloning strain | Site-directed mutagenesis and plasmid propagation | Takara |
| Strain | *E. coli* BL21(DE3) | Protein expression strain containing T7 RNA polymerase gene | Expression of wild-type and mutant *Bt*AS enzymes | Takara |
| Plasmid | pET28a-*Bt*AS | pET28a(+) carrying wild-type *Bt*AS gene | Template for mutagenesis and expression | This study |
| Plasmid | pET28a-BtAS-G374X variants | Mutant constructs of *Bt*AS at position 374 (site-saturation library) | Variant protein expression | This study |
| Primer | *Bt*AS-G374X_For | 5′-GCCATGACGATATCNNKTGGGGTCTGGAC-3′ | Site-saturation mutagenesis (forward) | This study |
| Primer | *Bt*AS-G374X_Re | 5′-GTCCAGACCCCAMNNGATATCGTCATG-3′ | Site-saturation mutagenesis (reverse) | This study |

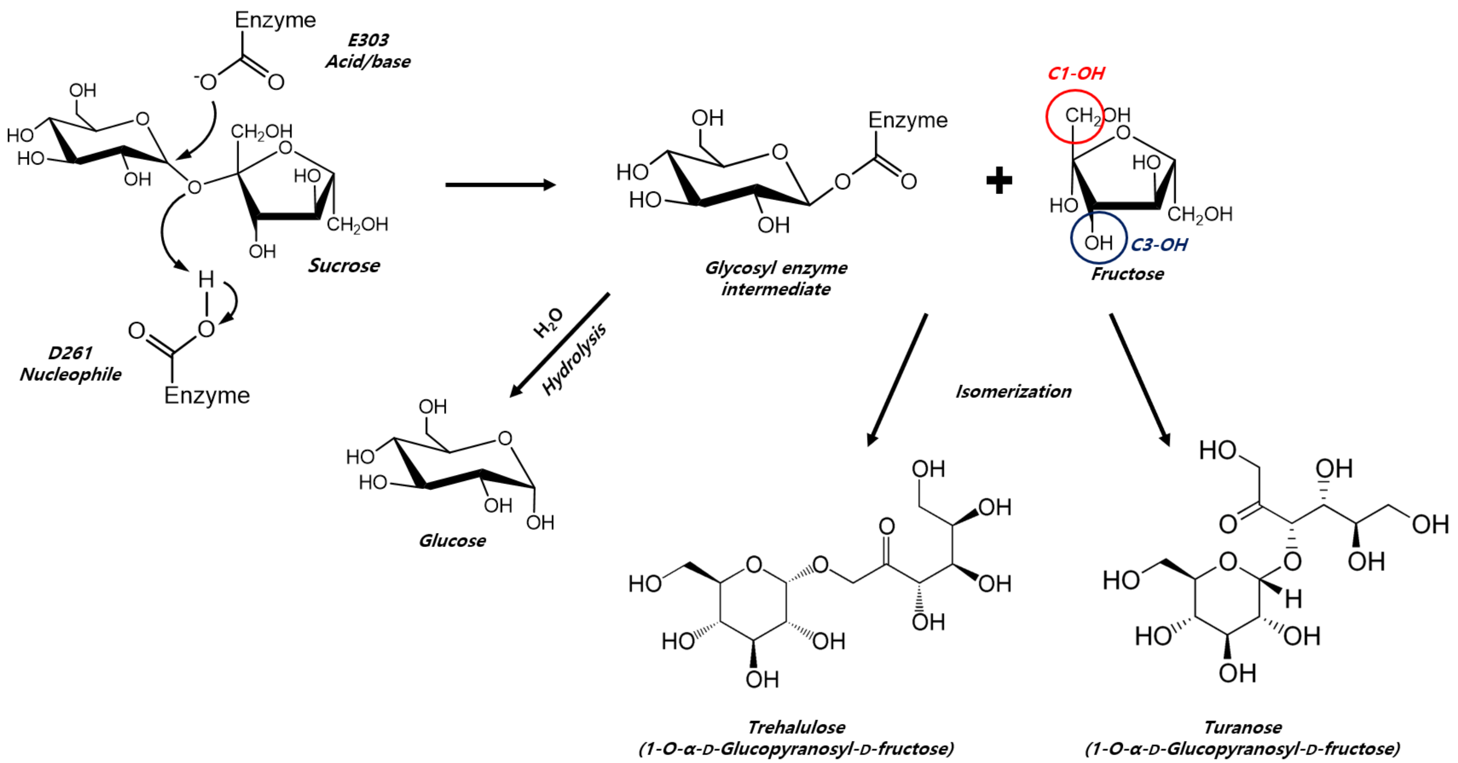


**Figure S1.** Proposed catalytic mechanism for sucrose isomerization by amylosucrase. The schematic illustrates the initial formation of the glycosyl-enzyme intermediate from sucrose and the subsequent nucleophilic attack by the fructose acceptor. Specifically, the reaction via the C1-OH group (highlighted in red) leads to the synthesis of trehalulose, whereas the reaction via the C3-OH group (highlighted in blue) leads to the formation of turanose.

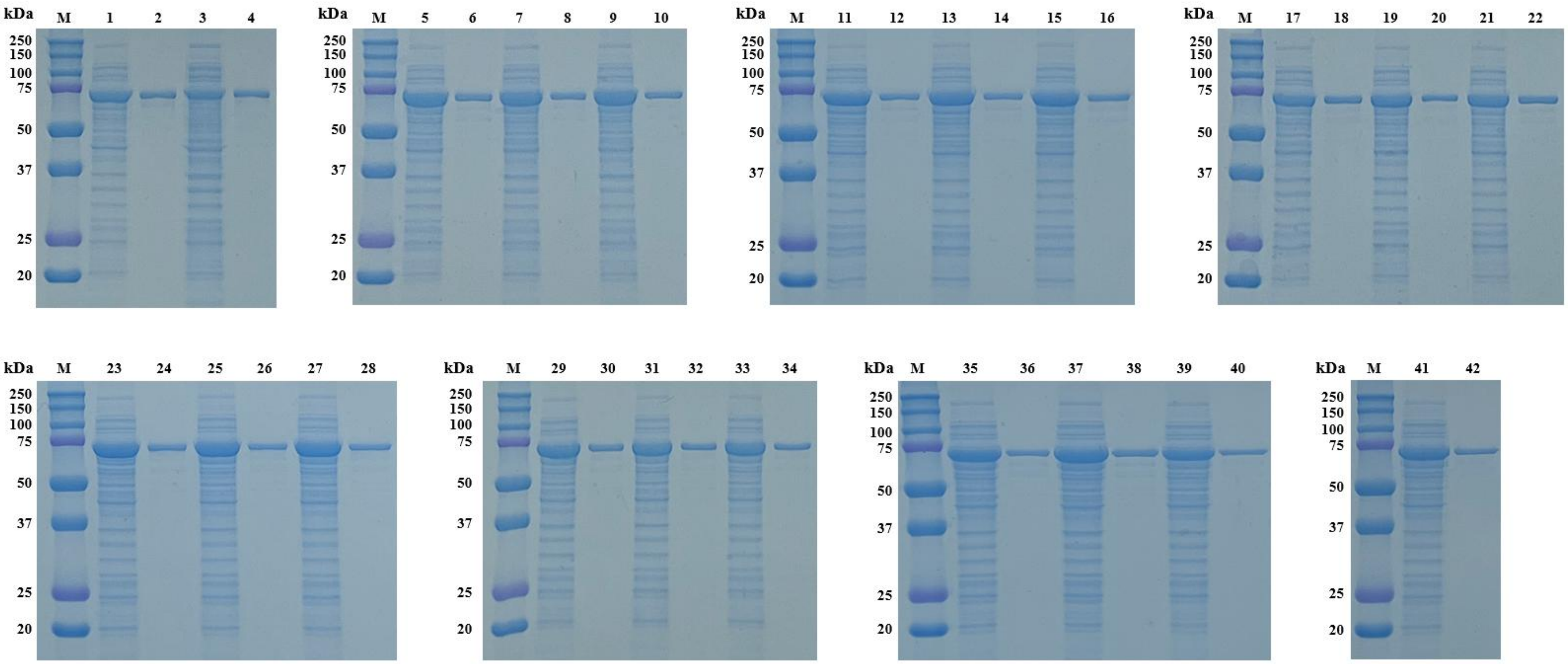


**Figure S2**. SDS-PAGE analysis of *Bt*AS variants with mutations at residue 374 expressed in E. coli BL21(DE3). M: Marker; 1-2: *Bt*AS wild-type (cell extract and purified enzyme); 3-4: *Bt*AS_G374H (cell extract and purified enzyme); 5-6: *Bt*AS_G374C (cell extract and purified enzyme); 7-8: *Bt*AS_G374Q (cell extract and purified enzyme); 9-10: *Bt*AS_G374T (cell extract and purified enzyme); 11-12: *Bt*AS_G374E (cell extract and purified enzyme); 13-14: *Bt*AS_G374D (cell extract and purified enzyme); 15-16: *Bt*AS_G374L (cell extract and purified enzyme); 17-18: *Bt*AS_G374M (cell extract and purified enzyme); 19-20: *Bt*AS_G374F (cell extract and purified enzyme); 21-22: *Bt*AS_G374A

(cell extract and purified enzyme); 23-24: *Bt*AS_G374R (cell extract and purified enzyme); 25-26: *Bt*AS_G374V (cell extract and purified enzyme); 27-28: *Bt*AS_G374I (cell extract and purified enzyme); 29-30: *Bt*AS_G374W (cell extract and purified enzyme); 31-32: *Bt*AS_G374K (cell extract and purified enzyme); 33-34: *Bt*AS_G374P (cell extract and purified enzyme); 35-36: *Bt*AS_G374Y (cell extract and purified enzyme); 37-38: *Bt*AS_G374N (cell extract and purified enzyme); 39-40: *Bt*AS_G374G (cell extract and purified enzyme); 41-42: *Bt*AS_G374S (cell extract and purified enzyme)

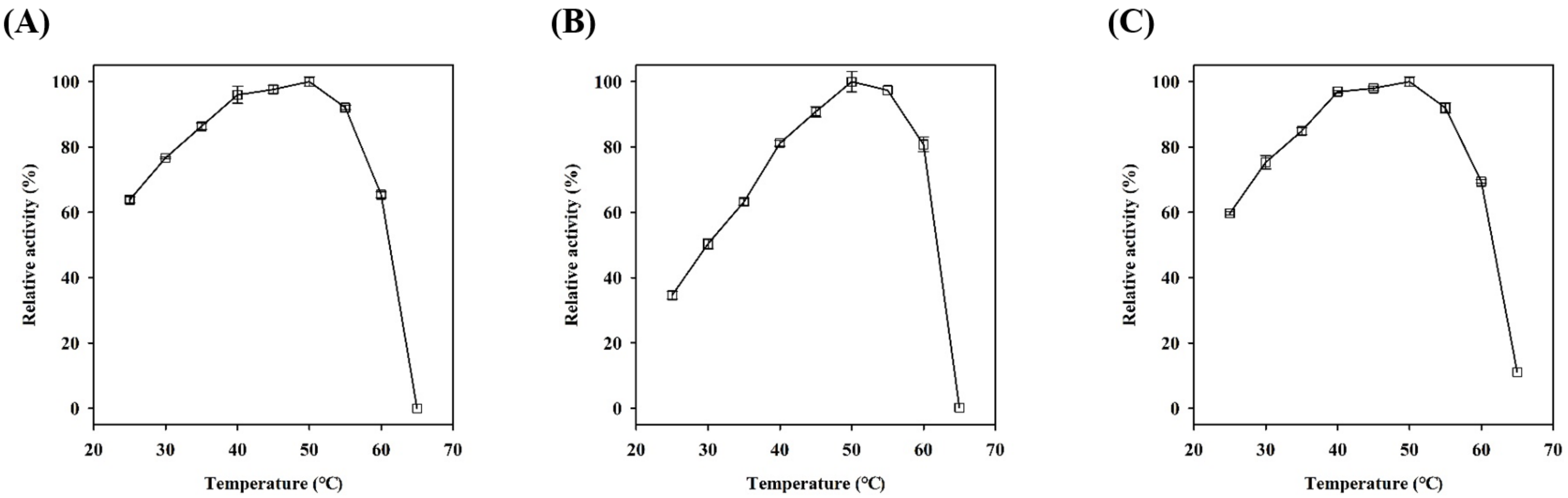


**Figure S3.** Effect of temperature on enzyme activity of *Bt*AS WT, G374H, and G374T variants. Reactions were performed at various temperatures for 30 min using 0.1 M sucrose as substrate in 50 mM sodium acetate buffer (pH 6.0). (A) *Bt*AS-WT; (B) *Bt*AS-G374H; (C) *Bt*AS-G374T

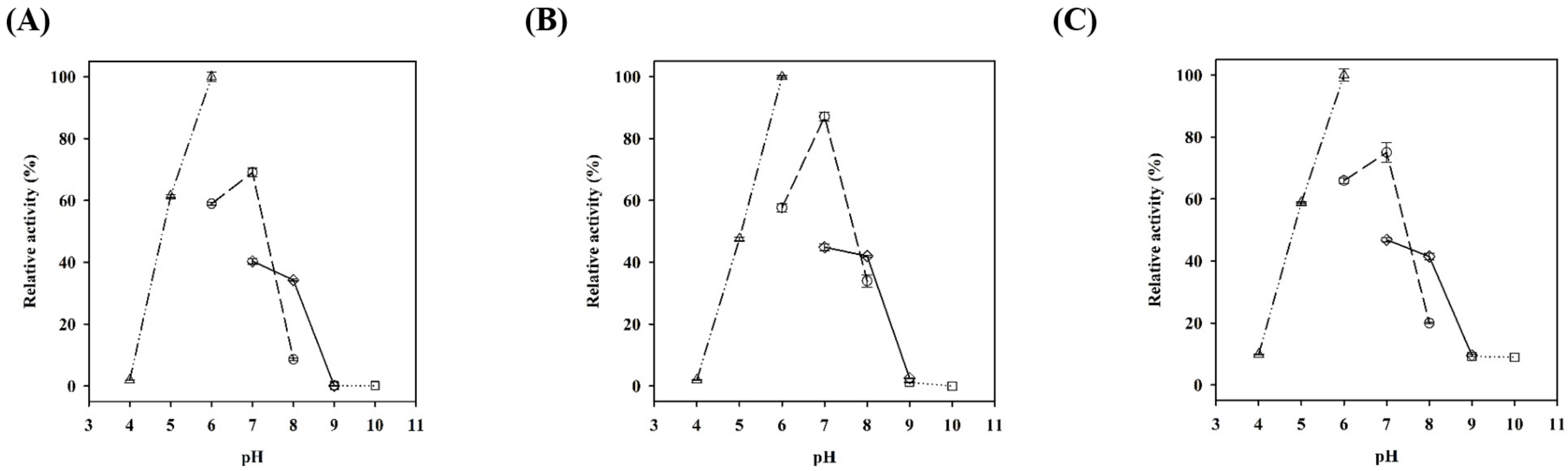


**Figure S4.** Effect of pH on enzyme activity of *Bt*AS WT, G374H, and G374T variants. Reactions were performed at 50°C for 30 min using 0.1 M sucrose as substrate. (A) *Bt*AS-WT; (B) *Bt*AS-G374H; (C) *Bt*AS-G374T. Symbols indicate different buffer systems: △, 50 mM sodium acetate; ○, 50 mM sodium phosphate; ◇, 50 mM Tris-HCl; □, 50 mM glycine-NaOH.

| States | *Bt*AS-WT | *Bt*AS-G374H | *Bt*AS-G374T |
| --- | --- | --- | --- |
| Enzyme-only (apo) | 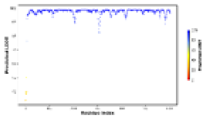 | 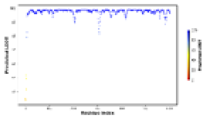 | 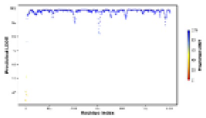 |
| Sucrose complex | 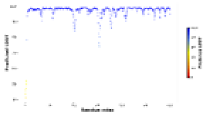 | 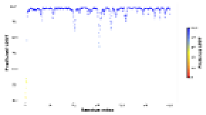 | 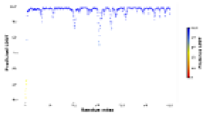 |
| Glycosyl - enzyme intermediate | 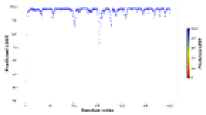 | 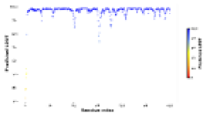 | 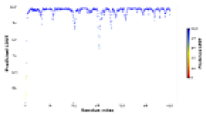 |
| Turanose complex | 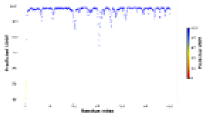 | 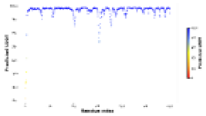 | - |
| Trehalulose complex | 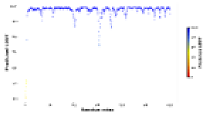 | - | 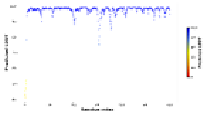 |

**Figure S5.** per-Residue confidence scores (pLDDT) for AlphaFold3-predicted structures of *Bt*AS WT, G374H, and G374T variants in different molecular states. The pLDDT scores indicate structural confidence, with higher values representing more reliable predictions. "-" indicates that the structure was not modeled for the corresponding state.

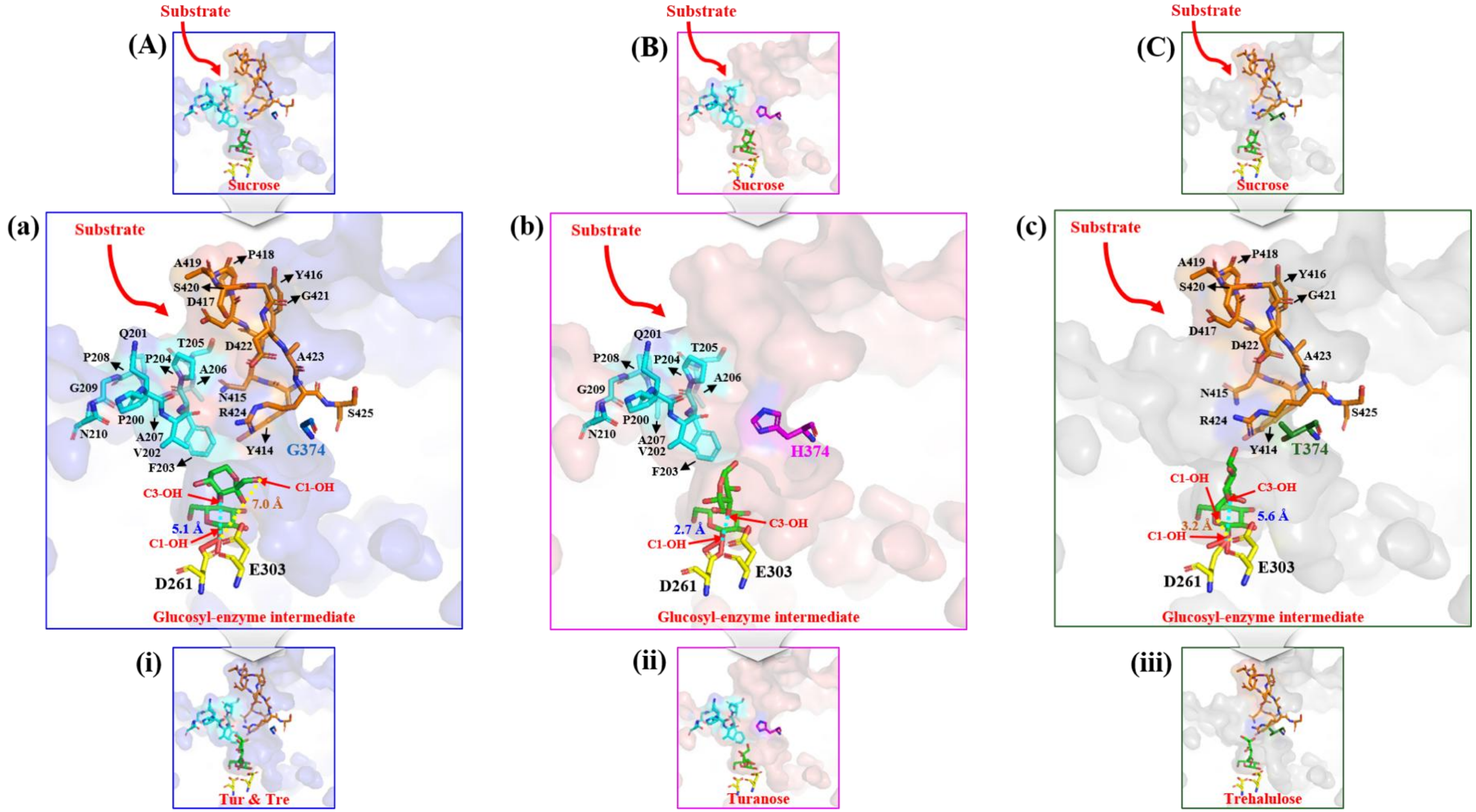


**Figure S5.** Three-dimensional structures of *Bt*AS variants showing different catalytic states. (A-C) Enzyme-sucrose complexes: (A) *Bt*AS-WT; (B) *Bt*AS-G374H; (C) *Bt*AS-G374T. (a-c) Glycosyl-enzyme intermediates with docked fructose: (a) *Bt*AS-WT; (b) *Bt*AS-G374H; (c) *Bt*AS-G374T. (i-iii) Product complexes: (i) *Bt*AS-WT with turanose; (ii) *Bt*AS-G374H with turanose; (iii) *Bt*AS-G374T with trehalulose.

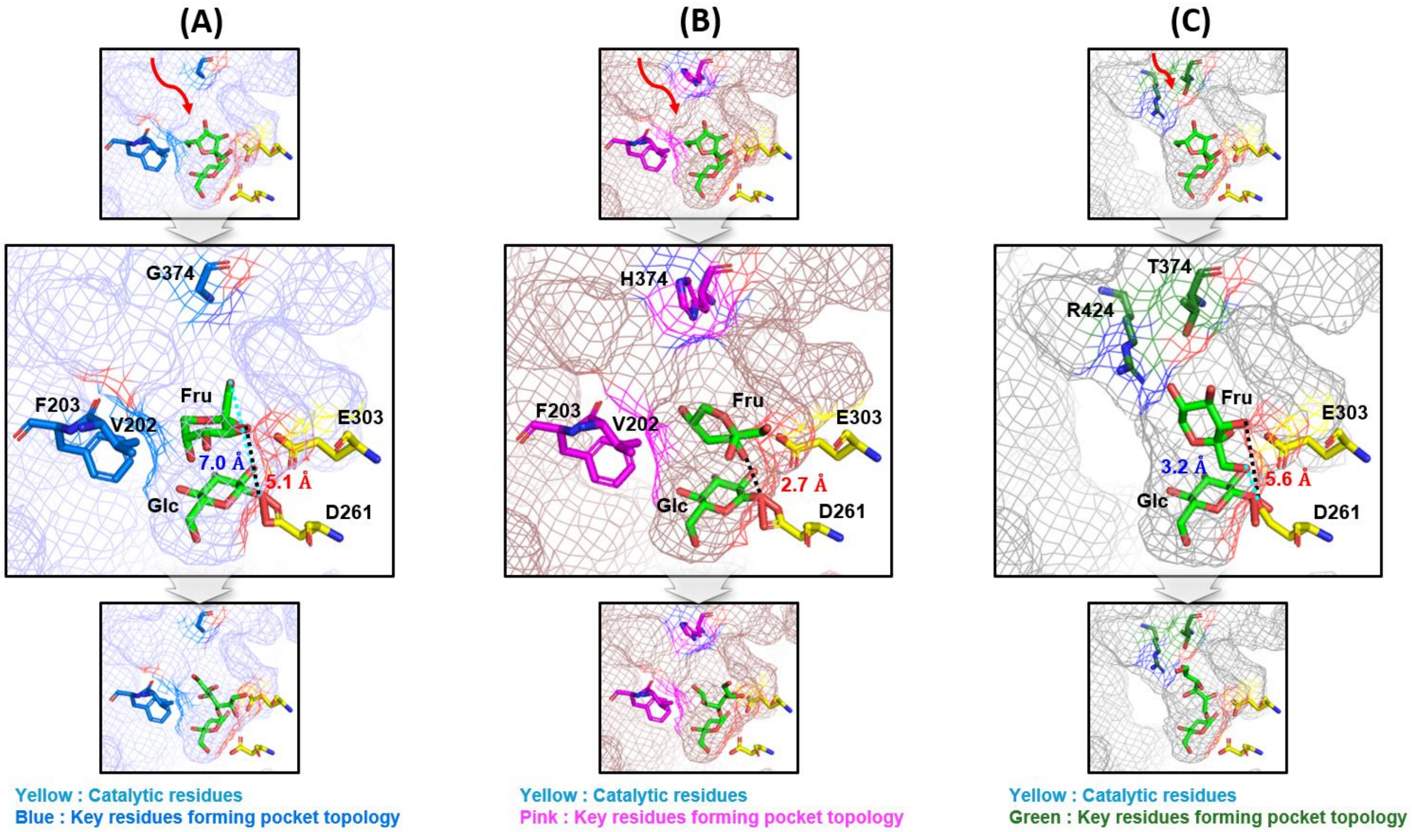


**Figure S7.** Substrate channel architecture during *Bt*AS catalysis. Channel/tunnel visualization for (A) WT, (B) G374H, (C) G374T. Upper: sucrose complex; Middle: intermediate with fructose; Lower: product complex. Mesh shows accessible volume. Yellow: catalytic residues; Blue: channel residues; Green/Magenta: position 374. G374H exhibits flexible channel for turanose formation; G374T shows structured pocket for trehalulose production.

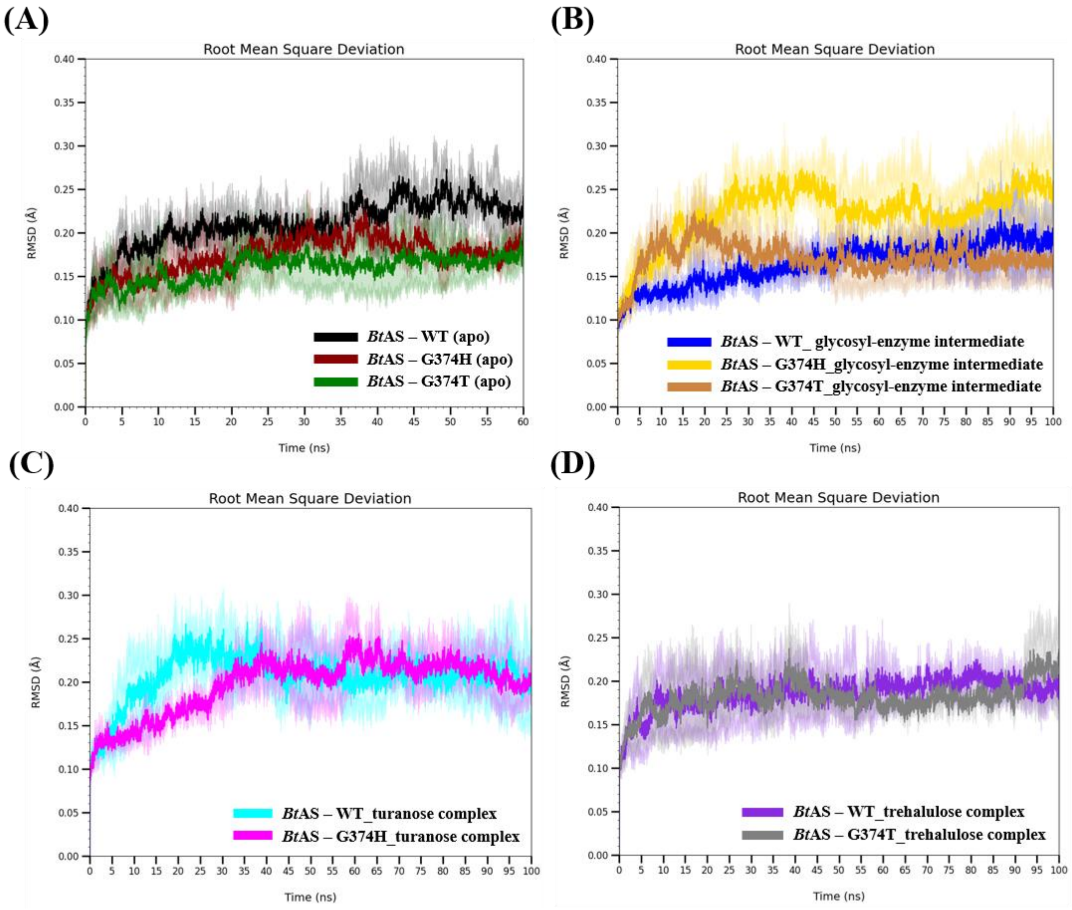


**Figure S8.** Root Mean Square Deviation (RMSD) analysis of *Bt*AS WT, G374H, and G374T variants over 100 ns of molecular dynamics simulations. (A) Enzyme-only (apo) state; (B) glycosyl-enzyme intermediate state with docked fructose; (C) enzyme complexes with turanose; (D) enzyme complexes with trehalulose.

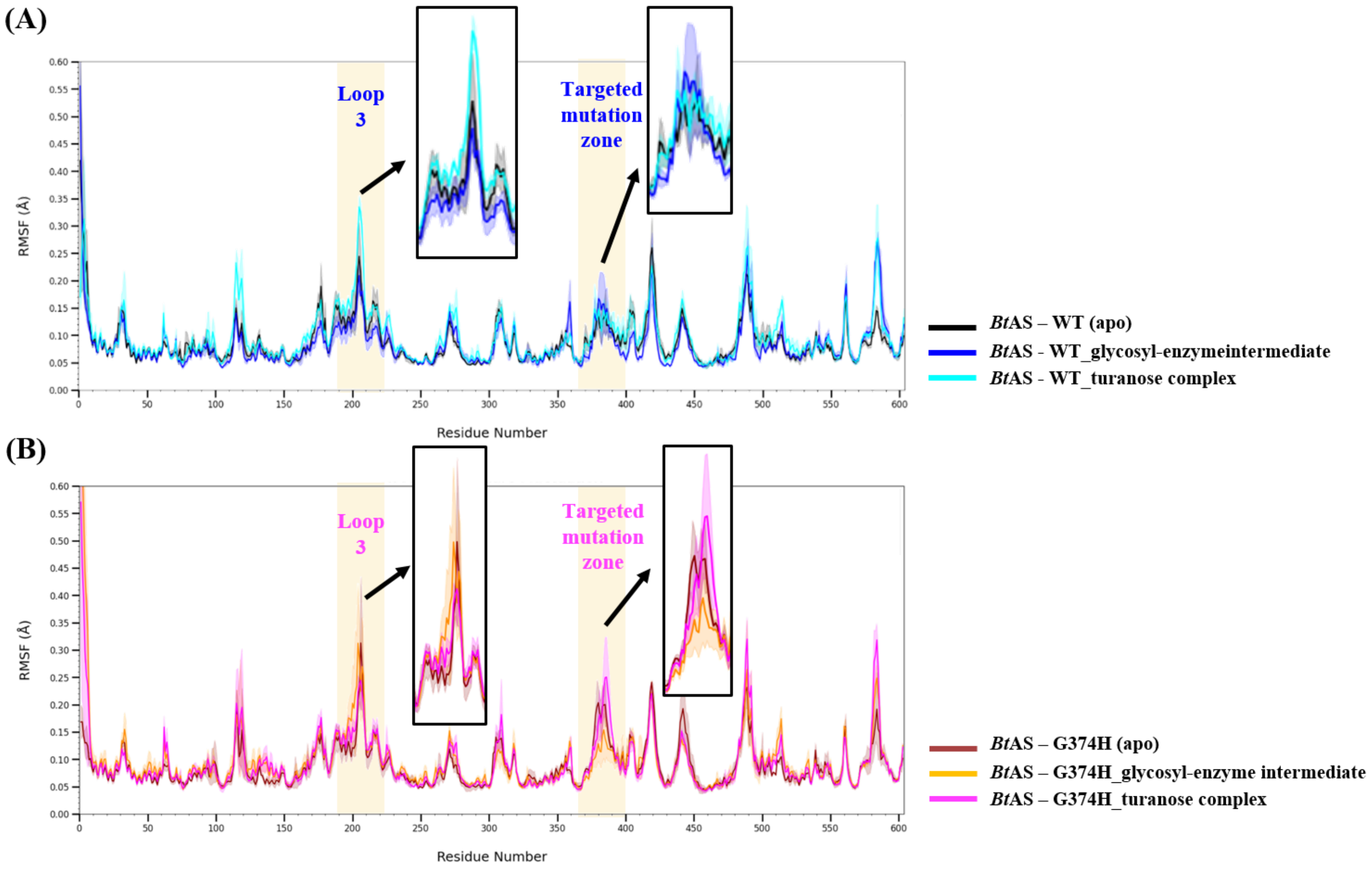


**Figure S9.** Root mean square fluctuation (RMSF) analysis of *Bt*AS WT and G374H variant over 100 ns of molecular dynamics simulations. (A) *Bt*AS-WT; (B) *Bt*AS-G374H. Key structural regions (Loop 3 and targeted mutation zone) are highlighted.

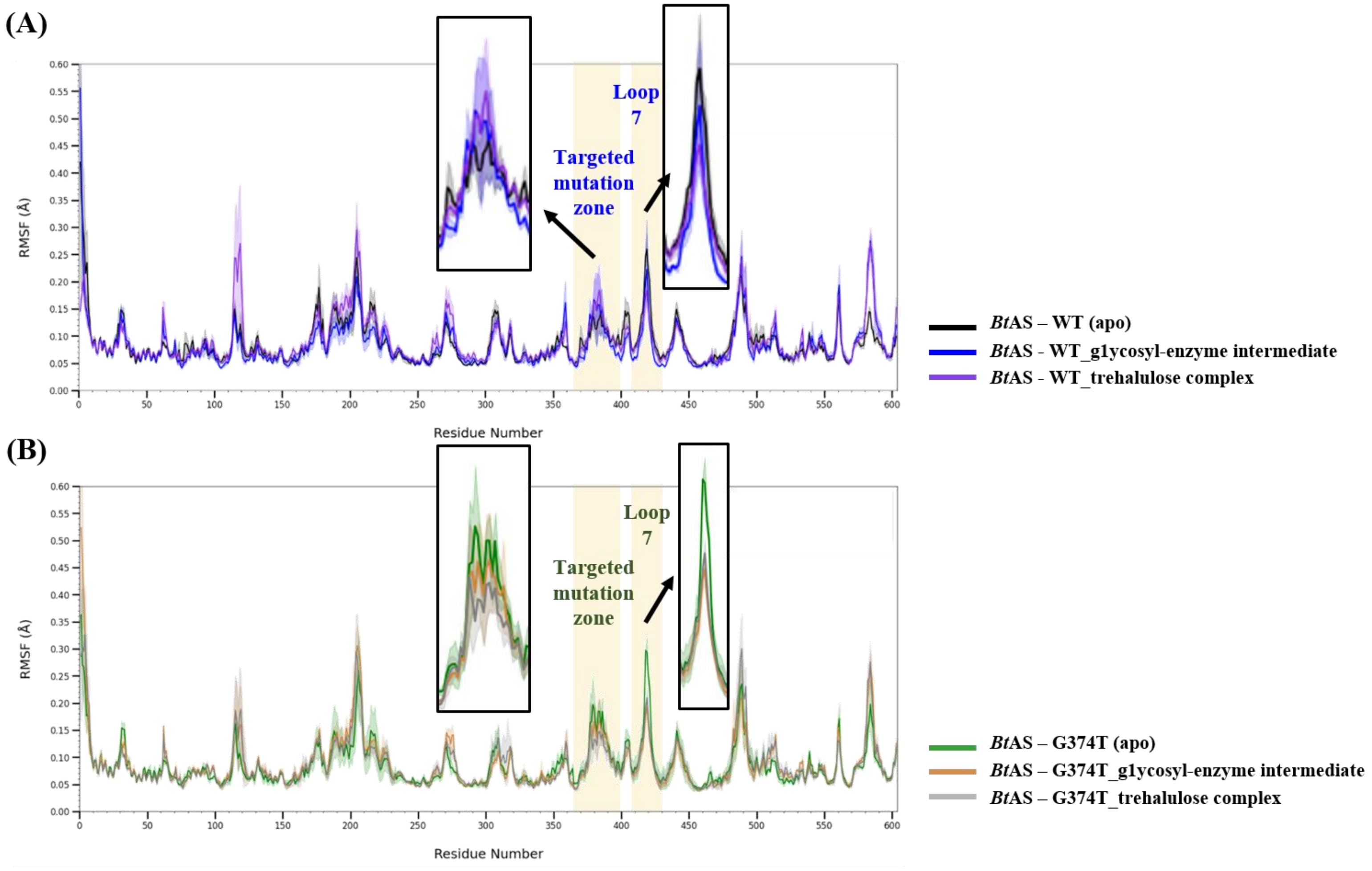


**Figure S10.** Root mean square fluctuation (RMSF) analysis of *Bt*AS WT and G374T variant over 100 ns of molecular dynamics simulations. (A) *Bt*AS-WT; (B) *Bt*AS-G374T. Key structural regions (Loop 7 and targeted mutation zone) are highlighted.

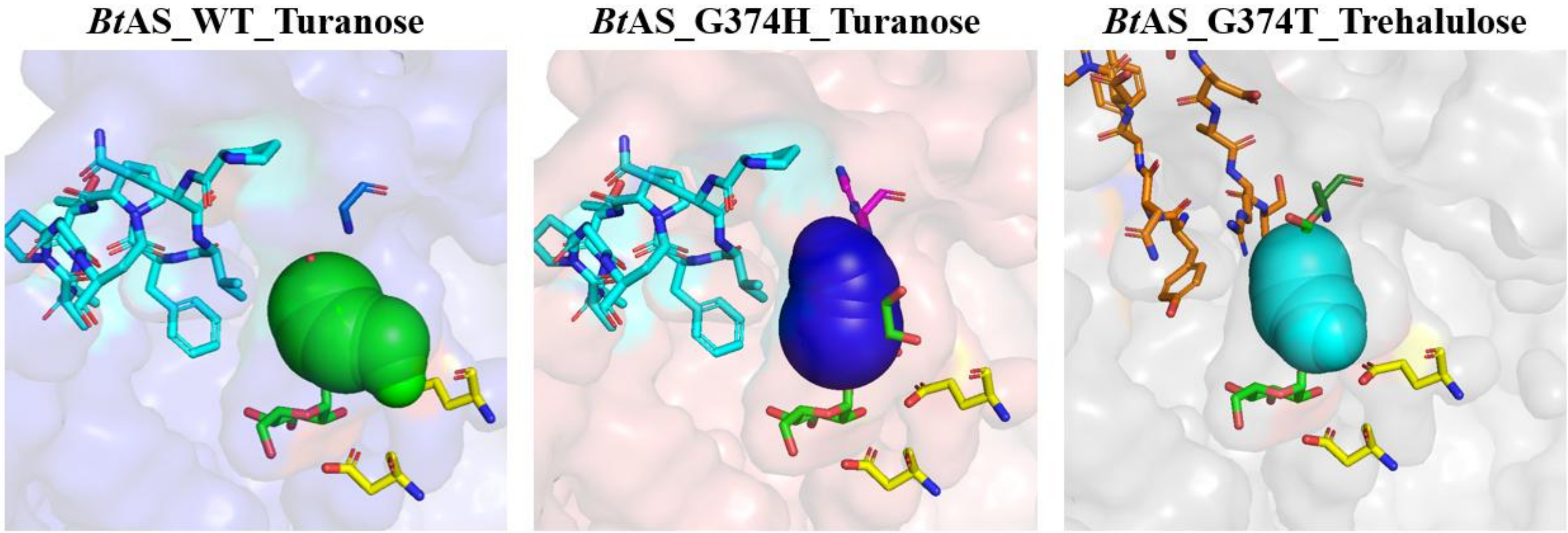


| protein | Avg_BR | Max_BR | Avg_L | Avg_C |
|---|---|---|---|---|
| ***Bt*AS_WT_Turanose** | **2.565** | **2.57** | **2.452** | **1.006** |
| ***Bt*AS_G374H_Turanose** | **1.994** | **1.99** | **4.826** | **1.125** |
| ***Bt*AS_G374T_Trehalulose** | **2.604** | **2.60** | **1.996** | **1.004** |

**Figure S11.** Quantitative analysis of product exit tunnels in WT and G374 variants. Detailed tunnel properties calculated using CAVER 3.0.3 are summarized in the table. The Average Bottleneck Radius (Avg_BR), Average Length (Avg_L), and Average Curvature (Avg_C) represent the physical dimensions and efficiency of the pathways for turanose and trehalulose dissociation.
